# Circumdatin D Exerts Neuroprotective Effects by Attenuating LPS-Induced Pro-Inflammatory Responses and Downregulating Acetylcholinesterase Activity *In Vitro* and *In Vivo*

**DOI:** 10.3389/fphar.2020.00760

**Published:** 2020-05-25

**Authors:** Chanjuan Zhang, Likun Hu, Dong Liu, Jian Huang, Wenhan Lin

**Affiliations:** State Key Laboratory of Natural and Biomimetic Drugs, Peking University, Beijing, China

**Keywords:** Alzheimer's disease, neuroprotective effects, pro-inflammatory response, acetylcholinesterase, circumdatin D

## Abstract

Alzheimer's disease (AD) is a prevalent neurodegenerative disorder with multifactorial causes, of which systemic inflammation may play a key role to promote neurodegeneration, and acetylcholinesterase (AChE) is a target protein to induce cholinergic transmission. Inhibitors toward inflammation and targeting AChE are regarded to promote cholinergic signaling of the central nervous system in AD therapy. During the search for neuroprotection agents from marine-derived compounds, seven circumdatin-type alkaloids from a coral-associated fungus *Aspergillus ochraceus* LZDX-32-15 showed potent inhibition against lipopolysaccharide (LPS)-induced nitric oxide (NO) production and activation of NF-κB report gene along with anti-AChE activities. Among the tested compounds, circumdatin D showed the most potent inhibitory effect against AChE activity and NO production. *In vivo* experiments using AD-like nematode models demonstrated that circumdatin D effectively delayed paralysis of CL4176 worms upon temperature up-shift *via* suppression of AChE activity and inflammatory-related gene expression. Moreover, circumdatin D interfered with inflammatory response by inhibiting the secretion of pro-inflammatory cytokines in LPS-induced BV-2 and primary microglia cells. Mechanistically, circumdatin D modulated Toll-like receptor 4 (TLR4)-mediated NF-κB, MAPKs and JAK/STAT inflammatory pathways in LPS-stimulated BV-2 cells, and protected primary neurons cells from LPS-induced neurotoxicity. Thus, circumdatin D is a potential agent for neuroprotective effects by the multi-target strategy.

## Introduction

Alzheimer's disease (AD) is a prevalent neurodegenerative disorder accompanied with progressive memory loss and cognitive functions impairment (Hardy and Selkoe, 2002). According to World Health Organization, AD affects 47.5 million individuals worldwide in 2015, and epidemiological data predict that over 115 million of people will be affected by 2050 (Hung and Fu, 2017). Pathological AD is marked by the deposition of amyloid-β (Aβ) plaques, formation of neurofibrillary tangles, as well as activation of microglia and astrocytes, which ultimately lead to neuronal dysfunction and death (Hardy and Selkoe, 2002). Up to date, five drugs including four acetylcholinesterase inhibitors and one *N*-methyl *D*-aspartate receptor antagonist, have been approved by FDA to treat AD. These drugs can only postpone the progression development of the disease, but fail to achieve a definite cure (Kumar and Singh, 2015; Briggs et al., 2016; Khoury et al., 2017; Behrens et al., 2018). Thus, discovery of new agents for a cure for AD is urgently in need. Considering the extraordinarily complicated pathogenesis of AD, simultaneous impact on multi-targets are regarded as an important therapeutic strategy for treatment of this disease. (de Freitas Silva et al., 2018; Li et al., 2018; Cummings et al., 2019; Mesiti et al., 2019).

Neuroinflammation process plays a pivotal role in the initiation and progression of various neurodegenerative diseases. Physiologically, neuroinflammation is originally a protective response in the brain, but an overreacting inflammatory response is harmful. In fact, it inhibits the neuronal regeneration (Heneka and Kummer, 2014; Schain and Kreisl, 2017). Microglia, the brain's resident immune cells, are pivotal for the neuroinflammatory response observed in AD. Under normal conditions, microglia protect the brain from pathogens and help to maintain homeostasis of the tissue. When pathologically insulted, either *via* endogenous or exogenous stimulations, microglia can transform to an “activated” state, that modified their shapes to enable their phagocytic functions and release a variety of pro-inflammatory or cytotoxic factors, such as nitric oxide (NO), tumor necrosis factor-α (TNF-α), interleukin-1β (IL-1β), interleukin-6 (IL-6), reactive oxygen species (ROS), prostaglandin E2 (PGE2) and cyclooxygenase-2 (COX-2) (Salter and Stevens, 2017; Cowan and Petri, 2018; Hansen et al., 2018). The accumulation of proinflammatory factors resulted in neighboring neuronal damage and degeneration. Damaged neurons subsequently release certain immune substances, aggravating the inflammatory neurotoxicity, consequently causing chronically irreversible neuroinflammation (Colonna and Butovsky, 2017; Dong et al., 2019; Liu et al., 2019). Accordingly, inhibiting inflammatory response of microglial cells and protection of neuronal cells from damage may potentially prevent the development of AD. Therefore, a potential therapeutic strategy for AD is regarded to discover agents for inhibiting microglia activation and controlling systemic inflammation.

Acetylcholine (ACh) is an important neurotransmitter which has been implicated in learning and memory processes. Cognitive decline in AD patients was associated with the deficiency of brain neurotransmitter Ach. Acetylcholinesterase (AChE) is a hydrolase that hydrolyzes acetylcholine into acetic acid and choline (Anand and Singh, 2013; Galimberti and Scarpini, 2016). The AChE levels elevated 20% in the plasma of AD patients when compared with those of age- and gender-matched controls (Sun et al., 2017). Inhibition of AChE prevents the breakdown of ACh and subsequently increases in ACh concentration and duration of action, which are considered to be clinically beneficial for AD patients. Thus, AChE inhibitors are widely used for the treatment of AD (Ferreira-Vieira et al., 2016). Currently, four of the five prescribed treatments for AD are AChE inhibitors. Apart from alleviation of some AD symptoms by promoting cholinergic signaling, these cholinergic drugs have weak efficacy to cure the disease (Ritchie et al., 2004; Li et al., 2016). Hence, it is important to develop new AChE inhibitors for the treatment of AD.

Nowadays, multi-targeting agents have been referred as an effective strategy for the treatment of multifactorial diseases like AD (de Freitas Silva et al., 2018; Cummings et al., 2019; Mesiti et al., 2019; Wang et al., 2019). Among several degenerative features that have been identified, neuroinflammation and cholinergic deficit are considered as the major contributing factors in the pathogenesis of AD. Thus, compounds with both AChE inhibition and anti-inflammatory qualities are attractive for the discovery of multi-targeted drugs acting with different mechanisms related to the disease. Natural products especially alkaloids are the prospective source for the discovery of new AChE inhibitor (Ng et al., 2015). Among naturally occurring alkaloids, circumdatins are a series of quinazoline benzodiazepine alkaloids isolated from coral-associated fungus *Aspergillus ochraceus*. Some of these alkaloids have been reported to have several biochemical activities such as cholecystokinin antagonism, inhibition of substance P and mitochondrial NADH oxidase production (Bock et al., 1986; Sun et al., 1994; López-Gresa et al., 2005; Chuan et al., 2009). In the process of seeking bioactive compounds derived from marine-associated fungi to assess anti-inflammation and the neuroprotective properties, a bioassay guided fractionation led to the isolation of seven circumdatin-type alkaloids, of which circumdatin D exhibited potent neuroprotective effects by the multi-target strategy, including the inhibitory effect toward AChE and interference with pro-inflammatory response *in vitro* and *in vivo*.

## Methods

### Chemicals and Materials

Dulbecco's modified Eagle medium (DMEM), α-minimum essential medium (α-MEM) and fetal bovine serum (FBS) were provided by Hyclone (Waltham, USA). 3-(4,5-Dimethylthiazol-2-yl)-2,5-diphenyltetrazolium bromide (MTT), acetylcholinesterase (AChE) from *Electrophorus electrics* (Electric eel) and lipopolysaccharide (LPS) (*Escherichia coli* 055: B5) were provided by Sigma Chemical Co. (St Louis, MO, USA). Enzyme-linked immunosorbent assay (ELISA) kits for IL-1β and TNF-α and Griess reagent for nitric oxide (NO) assay kit were provided by ExCell Bio (Shanghai, China). Antibodies for TLR4, MyD88, JNK, p-JNK, p38, p-p38, ERK, p-ERK, IκB, p-IκB, IKK, p-IKK, NF-κB, STAT3、p-STAT3、Jak2, p-Jak2, GAPDH and Histone H3 were purchased from Cell Signaling Technology (Danvers, USA). Antibodies for iNOS and COX-2 were provided by Abcam (Cambridge, UK).

### Isolation of Circumdatins From Fungus *A. ochraceus* LZDX-32-15

A gorgonian coral (LZDX-32)-associated fungus *A. ochraceus* LZDX-32-15 was collected from the South China Sea. Fungal strains were cultured on potato agar medium (PDA). The protocol for a large-scale fermentation refereed to the method reported in the literature (Hu et al., 2019). The fermented material was extracted successively with EtOAc (3 × 500 ml) to obtain EtOAc extract (3.46 g) under vacuum concentration. After removing fatty acids, the EtOAc extract (1.0 g) was subjected to a silica gel column eluting with dichloromethane-acetone (15:1) to yield 10 fractions (F1 to F10). A bioassay using Griess method showed F3 fraction to be the most active against the NO production in LPS-induced BV-2 cells. F3 (400 mg) was repeatedly separated on ODS (C_18_) column using MeOH–H_2_OH (1:5) to collect three subfractions (F31–F33). F31 (180 mg) was separated upon ODS column eluding with MeOH–H_2_OH (1:5) to obtain circumdatin C (2, 128 mg) and circumdatin J (**7**, 39 mg). F32 (120 mg) was purified by semi-preparative HPLC using MeOH–H_2_O (1:4) as a mobile phase to yield circumdatin F (1, 46 mg), circumdatin G (3, 34 mg), and circumdatin H (6, 28 mg). F33 (60 mg) followed the same protocol as for F32 to obtain circumdatin I (4, 20) and 2-hydroxycircumdatin C (**5**, 21 mg). Their structures were identified on the basis of spectroscopic data and comparison of the data with those of authentic samples (see **S1**).

### Cell Culture and Treatment

BV-2 cells (murine microglia cell line) was obtained from Peking Union Medical College, Cell Bank (Beijing, China) and maintained in DMEM containing 10% FBS. Cells were grown in a humidified atmosphere of 5% CO_2_ incubator at 37°C.

BV2 cells were seeded and incubated overnight, and followed by treatment with different doses of circumdatins for indicated period with or without LPS (1 μg/ml) stimulation. The cells without circumdatins and LPS treatment were used as the control group.

Primary microglia were prepared from the cortical tissue of neonatal (1–3 d) Sprague Dawley rats as Ni and Aschner (2010) described and primary cortical neurons were isolated from embryos of pregnant Sprague Dawley rats of E-18 (Zeng et al., 2012). All experimental manipulations with the animals was reviewed and approved by the animal experimental ethics committee of Peking University. The animals used in the work were performed in accordance with the Animal Care and Use Guidelines of Peking University.

### MTT Assay

*In vitro* cytotoxicity was detected using the MTT assay. Cells were treated with different concentrations of circumdatins in the presence or absence of LPS for 48 h. Then, MTT reagent was added to each well and the plates were further incubated at 37°C for 4  h. The absorbance was read at 570 nm using a microplate spectrophotometer (Thermo Scientifics, USA).

### Measurement of NO Production

The NO levels in culture media was determined using a Griess method. Cells were treated with different concentrations of circumdatins in the presence or absence of LPS for 24 h. After that, the supernatant fraction of the culture media was collected and mixed with the same volume of Griess reagent (1% sulfanilamide/0.1% naphthyl ethylene diamine dihydrochloride/2% H_3_PO_4_). Then, the absorbance was measured at 540 nm using a microplate spectrophotometer (Thermo Scientifics, USA). The concentration of nitrite was calculated from a calibration curve obtained from standards sodium nitrite solutions.

### ELISA Assay for TNF-α and IL-β

Cells were treated with different concentrations of circumdatin D in the presence or absence of LPS for indicated time. Then, the culture media were collected and centrifuged at 12,000*g*, 4°C for 20 min. The levels of TNF-α and IL-β in the supernatants were determined by ELISA kits according to the manufacturer's instructions.

### NF-κB Luciferase Reporter Assay

SW480-NF-κB cells were seeded at a density of 5 × 10^4^ cells/well in 24-well plates and incubated with circumdatins with or without LPS stimulation for 6 h. The κB-dependent luciferase reporter activity in cell extracts was monitored using an automated microplate reader.

### Evaluation of Acetylcholinesterase Inhibitory Activity *In Vitro* and *In Vivo*

AChE activities *in vitro* and *in vivo* were assessed by a modified Ellman's spectrophotometric method (Szwajgier, 2015). Briefly, AChE (80 μl) in phosphate buffer (Na_2_HPO_4_/NaH_2_PO_4_, pH 8.0) and compound (20 μl) diluted in same phosphate buffer were added to 96 microplates as the reaction mixture. The mixture was incubated at 37°C for 30 min before addition of 10 μl 5,5′-dithiobis (2-nitrobenzoic acid) (DTNB) (3 mM) and 10 μl acetylthiocholine iodide (ATCI) (15 mM). After incubation for 20 min, the absorbance was detected at 412 nm using a microplate spectrophotometer (Thermo Scientifics, USA). Galanthamine hydrobromide (1 µM) was used as positive control. The inhibitory rate of AChE was calculated, and the IC_50_ values were calculated by GraphPad Prism^®^ 5.0 software (GraphPad Inc., San Diego, USA).

### Affinity Detection Between Circumdatins and Acetylcholinesterase

The experiments for surface plasmon resonance (SPR) interaction were conducted at 25°C using the Biacore T200 system (GE Healthcare, Sweden). Recombinant AChE diluted in sodium acetate buffer (100 mM, pH 4.5) was used as the stock for the following experiments. CM5 sensor chips were coupled with AChE using an amine coupling kit (GE Healthcare, Buckinghamshire, UK). Thereafter, different concentrations of compounds were injected as analytes using PBS-P (10mM phosphate buffer with 2.7 mM KCl and 137 mM NaCl, 0.05% polysorbate 20, pH 4.5) as running buffer. For binding assays, the analytes were introduced with a 60 s contact time followed by a 60 s dissociation time at a flow rate of 45 μl/min. The surface was regenerated with 10 mM glycine (pH 3.0) for 30 s. The affinity constants were calculated using the 1:1 Langmuir binding model of Biacore evaluation software (T200 Version 1.0).

### Microglia-Neuron Co-Culture

For co-culture experiments, primary microglia cells were seeded on the upper insert of Transwell chambers (pore size 3 μm; Corning Costar, USA) in 24-well plates, and neurons were grown in the lower plate wells. Circumdatin D was added to primary microglia cells 1 h before LPS stimulation. After 48 h of coculture, the neurons in the bottom of 24-well plate were collected for the following experiments.

After microglia-neuron co-culture for 36 h, the neurons were washed twice with PBS and fixed with cold 4% paraformaldehyde for 30 min. Then, the cells were stained with 1% crystal violet solution for 15 min. Images were captured with an optical microscope. Five different fields were selected for each group for observing cellular morphology.

After microglia-neuron co-culture for 36 h, neurons were washed twice with PBS, fixed with cold 4% paraformaldehyde for 30 min and permeabilized with 0.1% Triton X-100 for 10 min. TUNEL staining was performed to detect apoptosis using *in situ* cell death detection Kit (Roche Diagnostics, Mannheim, Germany). Hochest 33342 staining was used to detect total cell nuclei. Images were taken and analyzed on a confocal microscopy (Leica, Germany). Six fields from each sample were randomly selected and analyzed.

### Immunocytochemistry Assay

BV2 cells were seeded on glass coverslips and treated with circumdatin D in the presence or absence of LPS for indicated time. After that, the cells were washed twice with PBS and fixed with cold 4% paraformaldehyde for 30 min. Then, the cells were permeabilized with 0.1% Triton X-100 for 10 min and blocked with 5% bovine serum albumin for 1 h. After incubation with primary antibodies overnight at 4°C, the cells were incubated with Alexa Fluor 488-conjugated secondary antibodies (Proteintech, USA) for 1 h at room temperature. Furthermore, Hochest 33342 was added to stain cell nuclei. Images were acquired and analyzed using a confocal microscope (Leica, Germany).

### Western Blot

Total cell proteins were lysed using cell extraction buffer containing phenylmethylsulfonyl ﬂuoride and protease inhibitor cocktail. The cytosolic and nuclear extracts were collected using the Nuclear and Cytosolic extraction kit. The protein extracts were separated on SDS-PAGE and transferred to PVDF membranes (Millipore). The membrane was blocked with 5% nonfat milk for 1 h at room temperature, and incubated with indicated primary antibodies overnight at 4°C. After washing, the membrane was incubated with secondary antibodies conjugated to horseradish peroxidase (Proteintech, USA) at room temperature for 1 h. Then, the proteins of interest were visualized utilizing a chemiluminescence (ECL) detection system. The relative optical densities were analyzed using the Image Master™ 2D Elite software.

### Reverse Transcription and Polymerase Chain Reaction (RT-PCR) Analysis

Total RNA was extracted using TRIzol reagent (Life Technologies, NY) and then reverse-transcribed into cDNA by reverse transcriptase (Invitrogen). mRNA expression of target genes iNOS, COX-2, IL-1β and TNF-α was quantified in comparison to housekeeping gene GAPDH using SYBR green detection method on ABI 7500 system (Applied Biosystems). The relative gene expression was calculated using comparative Ct (ΔΔCt) method. The following primers in **Table 1** were used in this study.

**Table 1:** Primer Sequences for Real-Time PCR.

iNOS	F: TAGGCAGAGATTGGAGGCCTTG	R: GGGTTGTTGCTGAACTTCCAGTC
COX-2	F: CAGGCTGAACTTCGAAACA	R: GCTCACGAGGCCACTGATACCTA
TNF-α	F: CAGGAGGGAGAACAGAAACTCCA	R: CCTGGTTGGCTGCTTGCTT
1L-1β	F: TCCAGGATGAGGACATGAGCAC	R: GAACGTCACACACCAGCAGGTTA
GAPDH	F: TGTGTCCGTCGTGGATCTGA	R: TTGCTGTTGAAGTCGCAGGAG

### *In Vivo Caenorhabditis elegans* Assay

The *in vivo* effects of circumdatin D on pro-inflammatory response and AChE activity were evaluated using *C. elegans* as animal model. Transgenic *C. elegans* CL4176 and *E. coli* OP50 were bought from the Caenorhabditis Genetics Center (University of Minnesota, Minneapolis, MN, USA). CL4176 strain become paralysis after up-shifting temperature from 16 to 25°C due to expressing human β-amyloid peptide in muscle cells. The nematodes were cultivated on nematode growth medium (NGM) plates (1.7% Agar, 0.3% NaCl, 0.25% peptone, 1 mM CaCl_2_, 1 mM MgSO_4_, 5 mg/L cholesterol, and 2.5 mM K_2_HPO_4_) with *E. coli* OP50 at 16°C. In paralysis assay, egg-synchronized CL4176 nematodes were incubated on solid NGM plates with *E. coli* OP50 at 16°C. When the age of the worms grew to L4 stage, nematodes were divided into four groups and exposed to appropriate concentrations of circumdatin D or vehicle control (0.4% DMSO) at 16 °C for 36 h. The temperature was then raised from 16 to 25°C, the number of paralyzed worms was counted under the microscope. The paralyzed worms were recognized for those with no response or only moved head when gently touched with a platinum wire. Each independent assay included three NGM plates with 30 worms for each one. Likewise, the AChE activity and RT-PCR assay was employed using similar conditions.

AChE activity of *C. elegans* was determined by the method as described (Xin et al., 2013). Briefly, approximately 1,000 nematodes with or without circumdatin D treatment were collected and then triturated mechanically in PBS (50 mM, pH 7.4) on ice. The lysate supernatant was collected by centrifugation and AChE activity in the supernatant was determined using modified Ellman method. The protein concentration was determined by BCA protein assay. AChE activity was expressed as U/mg proteins.

Related gene expression levels were detected as Xin et al. (2013) described. After treatment, CL4176 worms were collected and total RNA was extracted using TRIzol method. The cDNA was synthesized using cDNA synthesis kit (Invitrogen) according to manual instructions. The mRNA expression of target genes *viz*. F22E5.6, ZC239.12, ace-1 and ace-2 was quantified in comparison to housekeeping gene F23B2.13 using SYBR green detection method on ABI 7500 system (Applied Biosystems). The relative gene expression was calculated using comparative Ct (ΔΔCt) method. The following primers in **Table 2** were used in this study.

**Table 2:** Primer Sequences for Real-Time PCR.

ace-1	F: AGTGGGCTCCTGTTCGAGAA	R: CAATAGAAAATCACCATCGACAA
ace-2	F: CAATAATCAACTCATGGGCATCA	R: TTTTCGCGAGACGAAACGA
F22E5.6	F: TCCCCATACGAAACAACACA	R: CTCCTCCCAGCTTTTCCACAA
ZC239.12	F: CCAGAAGAATCCCCATACGA	R: TCCTCCTCCAACTTTTCCAAA
F23B2.13	F: CGCCGAAAATGAAATCAAAC	R: GGGCGTCGTACACCATCA

### Statistical Analysis

All data were expressed as mean ± standard deviations (SD) and analyzed using GraphPad Prism^®^ 5.0 software. Statistical analysis was carried out using a two-tailed Student's t-test when two groups were compared. A value of p <0.05 was considered as statistically signiﬁcant.

## Results

### Bioassay-Guided Separation of Circumdatins With Inhibition Pro-Inflammatory Response

**Figure 1:**
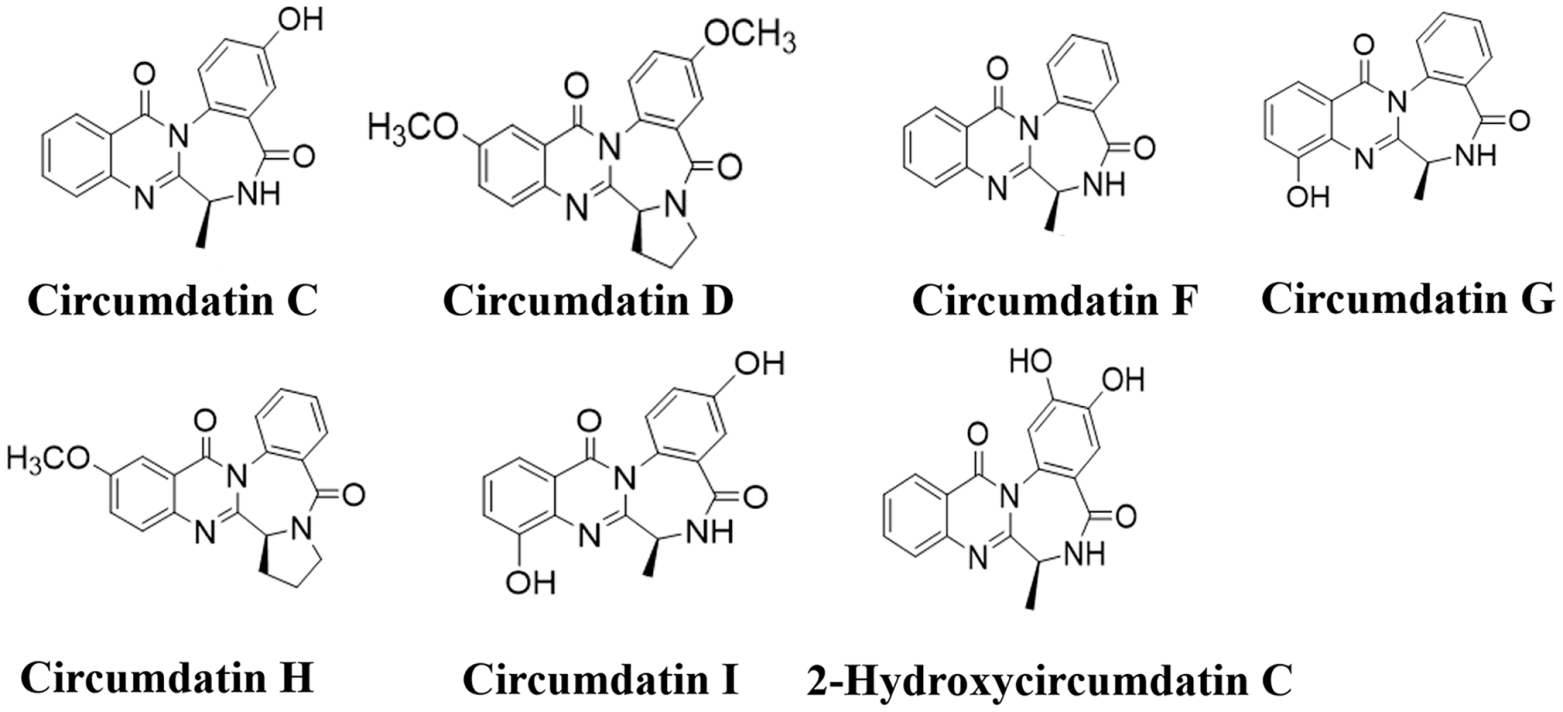
Structures of compounds 1–7.

Gorgonian coral (LZDX-32)-associated fungus *A. ochraceus* LZDX-32-15 was cultured in liquid fermentation. A bioassay guided fractionation showed that EtOAc fraction possessed inhibitory effects against LPS-induced nitric oxide production in BV-2 cells. Chromatographic separation of the bioactive fraction resulted in the purification of seven alkaloids, while they were identical to circumdatin C (1), circumdatin D (2), circumdatin F (3), circumdatin G (4), circumdatin H (5), circumdatin I (6), and 2-hydroxycircumdatin C (7) (**Figure 1** and **Figures S1–S15**), based on the spectroscopic data and comparison with those of authentic samples.

### Effects of Circumdatins on NO Production and NF-κB Dependent Luciferase Gene Expression

**Figure 2:**
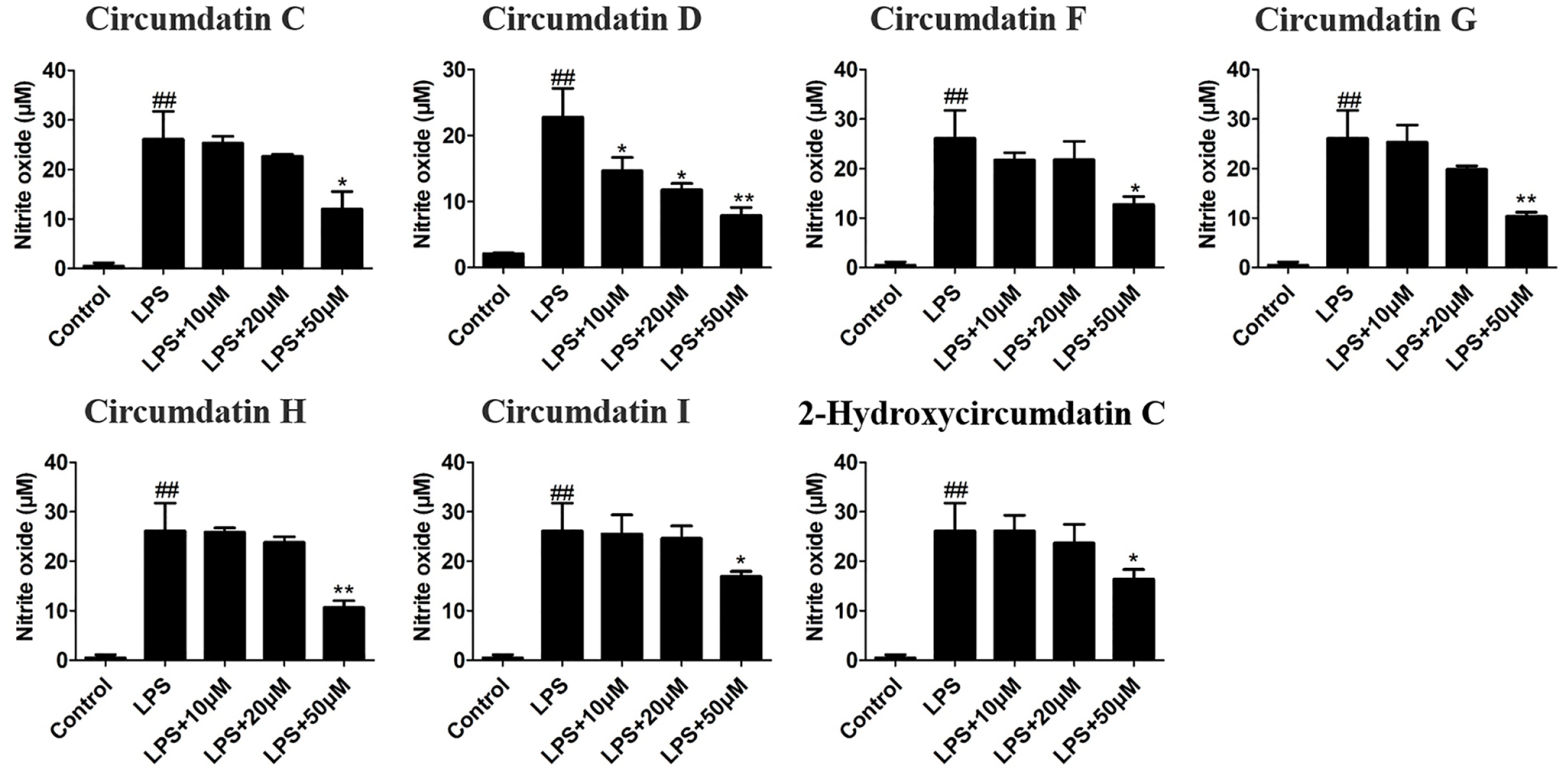
Effects of different concentrations of circumdatins on LPS-induced nitric oxide (NO) production in BV-2 cells. Cells were treated with circumdatin C, circumdatin D, circumdatin F, circumdatin G, circumdatin H, circumdatin I and 2-hydroxycircumdatin C in the presence or absence of 1 μg/ml LPS for 24 h. NO levels were measured by Griess method, Values represent the mean ± SD of three independent experiments (^#^compared with the control, *compared with LPS, *^/#^P < 0.05, **^/##^P < 0.01.

NO is a critical signaling molecule produced by enzyme iNOS at inflammatory sites, and high expression of NO is a significant feature of neuroinflammation and related disease conditions. Seven circumdatin-type alkaloids (**1**–**7**) showed inhibitory effects against LPS-stimulated NO production in BV-2 cells under non-toxic dose (**Figure S16**). As shown in **Figure 2**, circumdatins D and G exhibited significant reduction of NO levels in LPS-stimulated BV-2 cells with a dose-dependent manner (p < 0.05). Circumdatins C, F, H and I decreased NO production only in high dose. In addition, circumdatins (**1**–**7**) showed potential inhibitory effects toward NF-κB reporter gene expression (**Table 3**), of which circumdatin D was the most active with the IC_50_ value lower than others. As an important transcription factor in the inflammatory pathway, NF-κB plays an important role in the regulation of various inflammatory factors (Shih et al., 2015), while compounds with the inhibition of NF-κB activation may be active to reduce inflammatory response.

**Table 3:** Inhibitory Effects of Circumdatins toward NF-κB Activation and AChE Activity.

Compounds	NF-κB Activation	AChE Activity	AChE Affinity
	IC_50_ (μM)^a^	IC_50_ (μM)^b^	K_d_(M)^c^
circumdatin C	15.6 ± 2.3	13.9 ± 0. 7	8.5 × 10^−5^
circumdatin D	8.7 ± 1.3	2.4 ± 0.5	1.9 × 10^−6^
circumdatin F	11.8 ± 0.9	10.0 ± 1.9	2.4 × 10^−4^
circumdatin G	18.9 ± 2.1	15.8 ± 2.0	1.2 × 10^−4^
circumdatin H	33.3 ± 1.4	98.0 ± 5.7	9.5 × 10^−5^
circumdatin I	18.6 ± 1.9	11.6 ± 2.6	6.5 × 10^−5^
2-hydroxycircumdatin C	16.5 ± 1.9	5.6 ± 0.9	3.6 × 10^−6^
galanthamine		0.1 ± 0.2	4.9 × 10^−7^

a SW480 cells are stably transfected with NF-κB luciferase reporter gene. IC_50_ values of circumdatins against NF-κB activation in SW480 cells induced by LPS. Values were expressed as mean ± SD (n = 3). The reported values are the mean of at least three independent measurements.

b IC_50_ values of circumdatins against AChE enzyme activity tested using modified Ellman's colorimetric method. Values were expressed as mean ± SD (n = 3). The reported values are the mean of at least three independent measurements.

c Equilibrium dissociation constants (K_d_) values were determined by SPR analysis on AChE-circumdatins complexes.

### Effects of Circumdatins on AChE Inhibition

AChE inhibitory activity of circumdatins was examined by modified Ellman's method. Circumdatins moderately inhibited AChE activities with IC_50_ values ranging 2.4–98.1 μM (**Table 3**), comparing to the positive control galanthamine (IC_50_ 0.1 μM), a well-known AChE inhibitor. Particularly, circumdatin D is the most active compound among the tested compounds with an IC_50_ value of 2.4 μM. In SPR experiment, the capability of the affinity of circumdatins with AChE was in binding equilibrium dissociation constant (K_D_ = kd/ka). Among the tested compounds, circumdatin D had a lower K_D_ value than other circumdatins, indicating a higher affinity with AChE (**Table 3**). This result consists with the aforementioned enzyme inhibition for that circumdatin D possessed inhibitory activity, assigning it to be an AChE inhibitor.

### Circumdatin D Inhibited IL-1β and TNF-α Production in LPS-Induced BV-2 Cells

**Figure 3:**
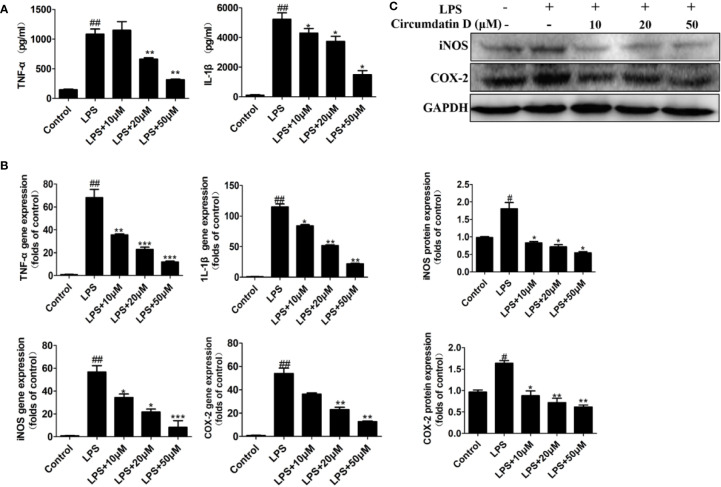
Circumdatin D downregulated inflammatory cytokines in LPS-induced BV-2 cells. **(A)** Cells were stimulated with 1 μg/ml LPS in the absence or presence of circumdatin D (10, 20, and 50 μM) for 6 h for measurement of TNF-α, and 8 h for measurement of IL-1β. The production of TNF-α and IL-1ß were measured by ELISA. **(B)** Cells were stimulated with 1 μg/ml LPS in the absence or presence of circumdatin D (10, 20, and 50 μM) for 24 h. Total RNA was prepared, and the mRNA levels of TNF-α, IL-1β, iNOS and COX-2 were determined using RT-PCR. GAPDH mRNA served as a control. **(C)** Cells were stimulated with 1 μg/ml LPS in the absence or presence of circumdatin D (10, 20, and 50 μM) for 24h. The protein expressions of iNOS and COX-2 were determined by Western blot assay. The data are represented as a mean ± S.D. from independent experiments performed in triplicate (^#^compared with the control, *compared with LPS, *^/#^P < 0.05, **^/##^P < 0.01, ***P < 0.001).

The effects of circumdatin D on the BV-2 cells cytotoxicity in the presence of LPS were detected by MTT assay. As shown in **Figure S17**, circumdatin D at the doses ranging 0–100 μM did not induce significant cellular death in BV-2 cells. Subsequently, the inhibitory effects of circumdatin D on pro-inflammatory cytokines secretion were detected. LPS alone (1 μg/ml) significantly increased the levels of TNF-α and IL-1β in BV-2 cells compared to that of the control, whereas circumdatin D suppressed LPS-induced TNF-α and IL-1β production in BV-2 cells with dose dependently (**Figure 3A**). In addition, circumdatin D also significantly decreased proinflammatory cytokines in LPS-stimulated BV-2 cells in the mRNA levels (**Figure 3B**).

### Circumdatin D Suppressed iNOS and COX-2 Expression in LPS-Induced BV-2 Cells

Nitric oxide synthase (iNOS) and cyclooxygenase-2 (COX-2) are two important enzymes in the inflammatory response. Using q-PCR analysis, circumdatin D showed dose-dependently decreasing the expression levels of both iNOS and COX-2 in LPS-induced BV-2 cells (**Figure 3B**). Furthermore, LPS significantly increased the protein expression of iNOS and COX-2 (**Figure 3C**), while circumdatin D reduced LPS-induced COX-2 and iNOS expression dose-dependently. These findings indicated that the anti-inflammatory activity of circumdatin D was induced by the inhibition of pro-inflammatory cytokine expression.

### Circumdatin D Protects Neurons Through Inhibition of Microglial Activation and Decreasing Acetylcholinesterase Activity

**Figure 4:**
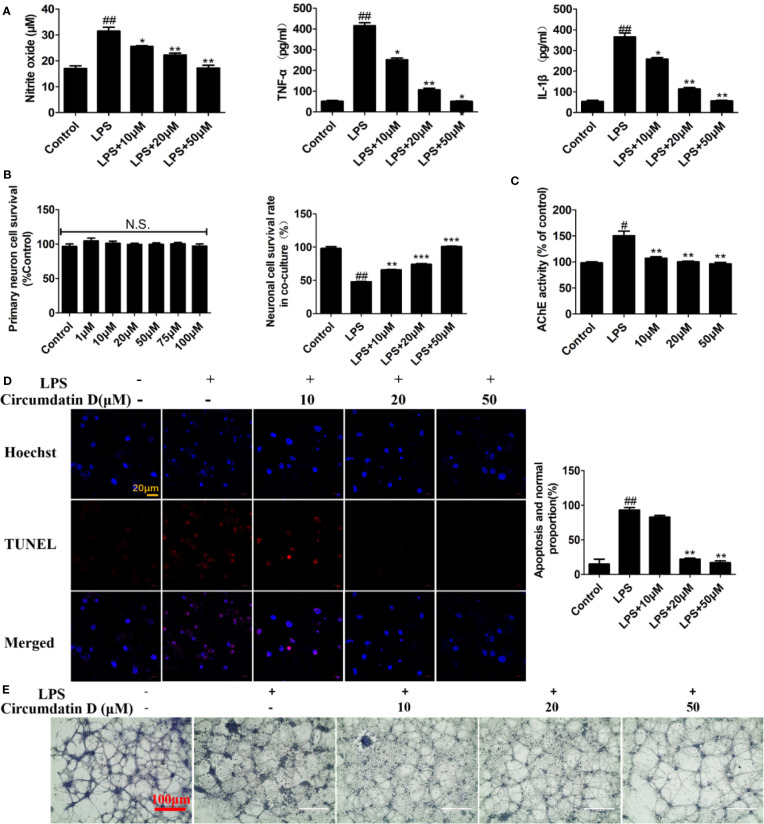
Circumdatin D prevented inflammation-induced neuronal death in microglial-neuronal co-culture and decreased acetylcholinesterase activity. **(A)** Primary microglia cells were stimulated with 1 μg/ml LPS in the absence or presence of circumdatin D (10, 20, and 50 μM) for measurement of NO, TNF-α, and IL-1β. NO levels were measured by Griess method. The production of TNF-α and IL-1ß were measured by ELISA. **(B)** Primary neuron cell was treated with or without circumdatin D (1–100 μM) in the absence or presence of 1 μg/ml LPS for 48 h by MTT assay. **(C)** Primary neurons were stimulated with 1 μg/ml LPS in the absence or presence circumdatin D (1–100 μM) for 48 h and AChE enzyme activity were tested by modified Ellman's spectrophotometric method. **(D)** Microglial–neuronal co-cultures were treated with 1 μg/ml LPS in the absence or presence of circumdatin D for 48 h, and then apoptotic neuronal cells were determined by TUNEL assay. Data were expressed as the ratio of the number of TUNEL-positive cells to the number of Hoechst-positive cells. **(E)** Microglial-neuronal co-cultures were treated with 1 μg/ml LPS in the absence or presence of circumdatin D for 48 h, and then crystal violet staining was performed to observe changes in morphology. Values represent the mean ± SD of three independent experiments (^#^compared with the control, *compared with LPS, *^/#^P < 0.05, **^/##^P < 0.01, ***P < 0.001. N.S., no significant differences from the control cells).

Since inflammation is associated with neurodegeneration during AD process, the neuroprotective effect of circumdatin D on activated primary microglia-induced neuronal cell death was investigated. In initial experiment, circumdatin D was added to primary microglial cultures in both untreated cells and LPS-stimulated cells. MTT data revealed that circumdatin D was low toxicity in these cultures within the doses of 1–100 μM (**Figure S18**). To assess the cytokine secretion in the supernatants of cultured microglial cells, LPS (1.0 μg/ml) induced primary microglia cells were treated with or without circumdatin D (10, 20 and 50 μM) in indicated times, and then inflammation mediators were quantified by appropriate assay kit. As shown in **Figure 4A**, circumdatin D significantly decreased the production of NO, TNF-α and IL-1β that were stimulated by LPS, showing the similar data as found in BV-2 cells. Subsequently, the neuroprotective effect of circumdatin D in neurons-microglia co-culture system was tested. As a preliminary assessment, incubating primary neuron-enriched cells with circumdatin D for 48 h was performed. MTT assay showed circumdatin D with no direct toxic to neurons in the tested doses (**Figure 4B**). In the co-culture system, primary microglia treated with LPS alone caused neuron death in a high level. This illustrated that LPS-activated microglia were able to induce neuron death by secreting pro-inflammatory cytokines and then migrating through the insert. Notably, treatment of primary microglia with circumdatin D prior to LPS stimulation reduced neuron death in the co-cultures as detected by MTT assay (**Figure 4B**). These data suggested that circumdatin D possessed cytoprotective effect by inhibiting microglial activation. Furthermore, a TUNEL assay was performed to examine cell apoptosis. In microglial-neuronal co-cultures, stimulation of primary microglia with LPS alone significantly enhanced neurons apoptosis as evidenced by an increase of the ratio of TUNEL-positive cells as compared to control (**Figure 4D**). Circumdatin D significantly reduced the numbers of TUNEL-positive cells and improved the LPS-induced survival. These data further confirmed the cytoprotective effect of circumdatin D on primary cortical neurons. In addition, crystal violet staining was used to observe cell morphology. **Figure 4E** clearly showed that neurons suffered inflammation-associated injury as obvious neurite loss and cleavage after LPS stimulation for 48 h, and circumdatin D markedly reversed the neuronal injury. These results suggested that circumdatin D exerted its neuroprotective effects, at least in part, *via* reducing the production and secretion of inflammatory mediators from the microglia.

Increasing the level of AChE led to acetylcholine deficit in AD, while the experimental data showed that the LPS-treated primary cortical neurons resulted in a significantly increasing AChE activity compared to the control group. Circumdatin D markedly reduced the LPS-induced AChE activities in primary neuronal cells (**Figure 4C**), suggesting circumdatin D directly inhibited AChE activity in neurons.

### Circumdatin D Attenuates Paralysis of Transgenic *C. elegans* Upon Temperature-Upshift

**Figure 5:**
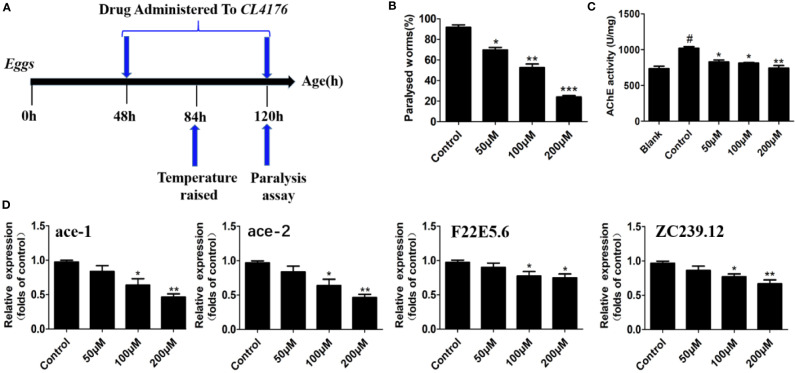
Circumdatin D delayed nematode paralysis upon temperature up-shift *via* suppression AChE and inflammatory-related gene expression in CL4176 transgenic *C. elegans* strains. **(A)** Timeline illustrating the time at which the temperature was raised from 16 to 25°C in egg-synchronized CL4176 worms, when worms were fed on circumdatin D-containing NGM medium and when the paralysis assay was scored. **(B)** The paralysis assays were quantified in the transgenic CL4176 strain treated with or without different concentrations of circumdatin D. Egg-synchronized CL4176 worms were incubated at 16°C for 48 h, treated with appropriate concentrations of circumdatin D at 16 °C for 36 h, then up-shifted to 25°C, and harvested at 36 h after temperature being up-shifted. Data are shown as percentages ± SD of worms paralyzed (n = 90, three independent assays). **(C)** Effect of circumdatin D on AChE activities, as measured by modified Ellman's spectrophotometric method in CL4176 transgenic *C. elegans*. Data are normalized with respect to control, and values are the mean ± SD of three independent experiments. **(D)** Real-time qRT-PCR analysis of AChE and inflammation -associated gene expression in CL4176 transgenic *C. elegans*. Total RNA was isolated from worms 36 h post temperature up-shift and subjected to DNase digestion. The gene expression levels were normalized to F23B2.13 mRNA. Values represent the mean ± SD of three independent experiments (^#^compared with blank, *compared with the control, *P < 0.05, **P < 0.01, ***P < 0.001).

As an ideal model organism, *C. elegans* has been widely utilized to assess the efficacy of anti-AD drug candidates (Lublin and Link, 2013). In the present work, the *in vivo* effect of circumdatin D on AD-like symptom was evaluated using a transgenic *C. elegans* CL4176 as a model organism. C4176 nematodes become paralyzed owning to the expression of a heat-induced human Aβ_1–42_ gene in the muscle cells, resulting in the production of inflammation cytokines and AChE elevation (Xin et al., 2013). Firstly, the toxicity of circumdatin D on CL4176 nematodes in 24 and 48 h was detected (**Figure S19**), showing no toxicity on the normal survival of the nematode at tested dose. **Figure S20** showed the paralysis time course for the non-treated CL4176 nematode. *C. elegans* displayed the paralysis phenotype during 24 h when temperature elevated from 16 to 25°C and the majority of the worms were paralyzed or die by 36 h. Therefore, 36 h post the temperature up-shift was used for all of the following experiments. To study protective effect of circumdatin D on CL4176 nematode, the percentage of paralyzed worms were recorded at 36 h after the temperature upshift according to the timeline (**Figure 5A**). Feeding with 50, 100, 200 μM of circumdatin D significantly reduced paralysis by about 24.9, 42.8 and 74.8% respectively (**Figure 5B**). This observation suggests that circumdatin D was potential to rescue paralysis in CL4176 nematodes. Upon temperature upshifted, the AChE activity was elevated in CL4176 nematodes. Circumdatin D significantly attenuated these changes in a dose-dependent manner (**Figure 5C**), indicating that circumdatin D is able to inhibit AChE *in vivo*. To delineate the mode of action, the expression levels of AChE genes (ace-1 and ace-2) and inflammation related gene (F22E5.6 and ZC239.12) were tested by qRT-PCR in CL4176. Circumdatin D inhibited temperature raise-induced AChE and inflammation-associated gene expressions respectively (**Figure 5D**), suggesting that circumdatin D alleviating paralysis in CL4176 was due to its capacity to inhibit AChE elevation and down-regulate inflammation gene expressions.

### Effect of Circumdatin D on the TLR4 and MyD88 Expression in LPS-Induced BV-2 Cells

**Figure 6:**
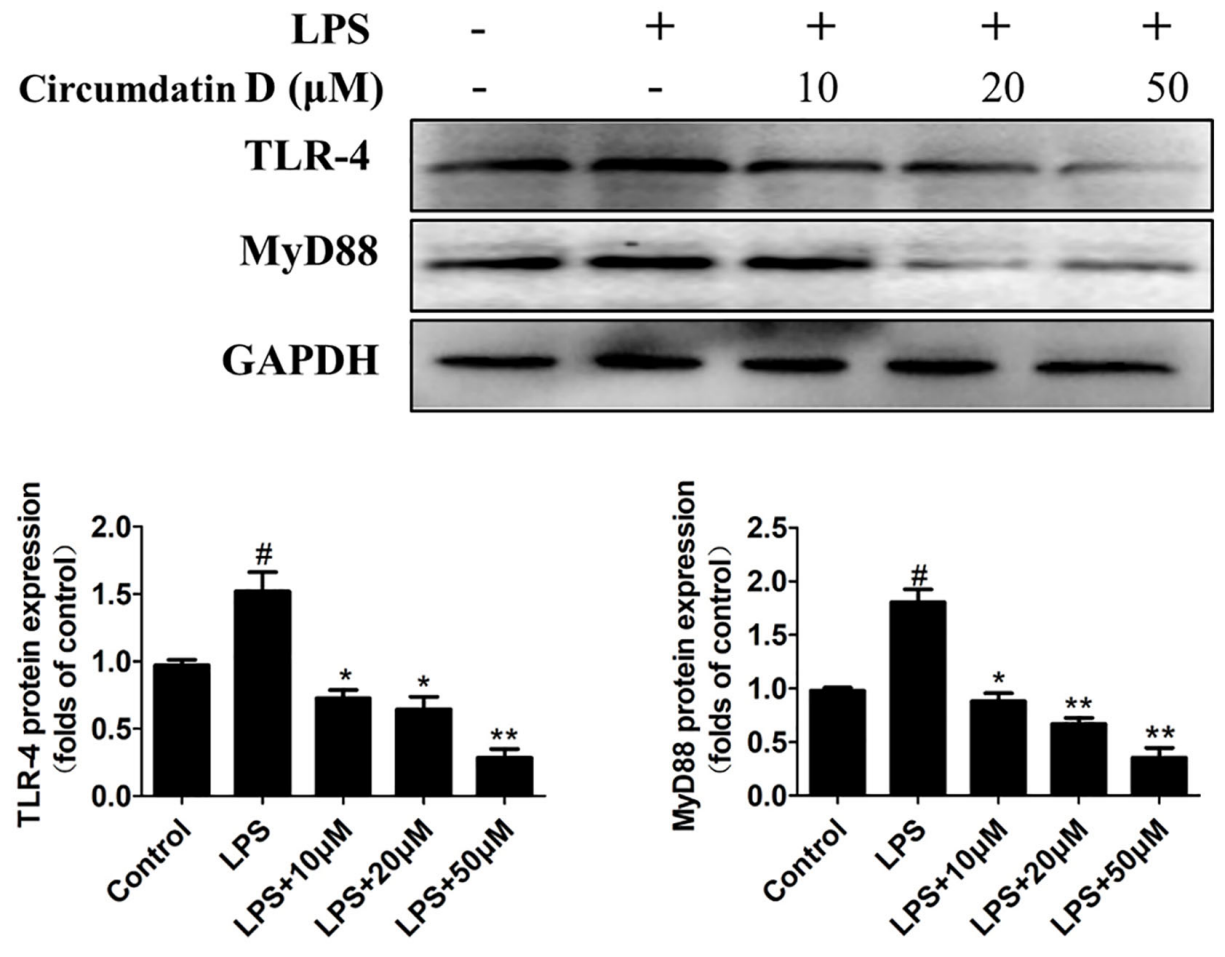
Circumdatin D inhibition of TLR4 and MyD88 protein expression in LPS-induced BV-2 cells. Cells were stimulated with 1 μg/ml LPS in the absence or presence of circumdatin D (10, 20, and 50 μM) for 6 h, cell lysates were prepared and subjected to western blot analysis for TLR4 and MyD88 expression. Values represent the mean ± SD of three independent experiments (^#^compared with the control, *compared with LPS, ^*/#^P < 0.05, ^**^P < 0.01).

As the major LPS receptor, Toll-like receptor 4 (TLR4) plays an important role in activation microglial through regulation of downstream NF-κB and MAPK signaling pathway (Wang et al., 2014; Cho et al., 2016; Rahimifard et al., 2017; Shabab et al., 2017). The effect of circumdatin D on LPS-induced TLR4 expression was examined to elucidate its anti-inflammatory mechanism. As shown in **Figure 6**, LPS enhanced TLR4 expression in BV-2 cells compared with untreated control. However, circumdatin D dramatically reversed the LPS-induced TLR4 expression. Moreover, Myeloid differentiation primary response protein 88 (MyD88) is an important adapter protein that could transduce signals from TLR4 to downstream NF-κB and MAPK signaling pathway and active inflammatory response. In LPS-treated BV-2 cells, MyD88 expression was increased, while circumdatin D suppressed the MyD88 expression (**Figure 6**). These results demonstrated that inhibition of pro-inflammatory response by circumdatin D was related to its regulation of TLR4 and MyD88 expression.

### Circumdatin D Attenuated LPS-Induced NF-κB, STAT3 Activation and MAPK Phosphorylation in BV-2 Cells

**Figure 7:**
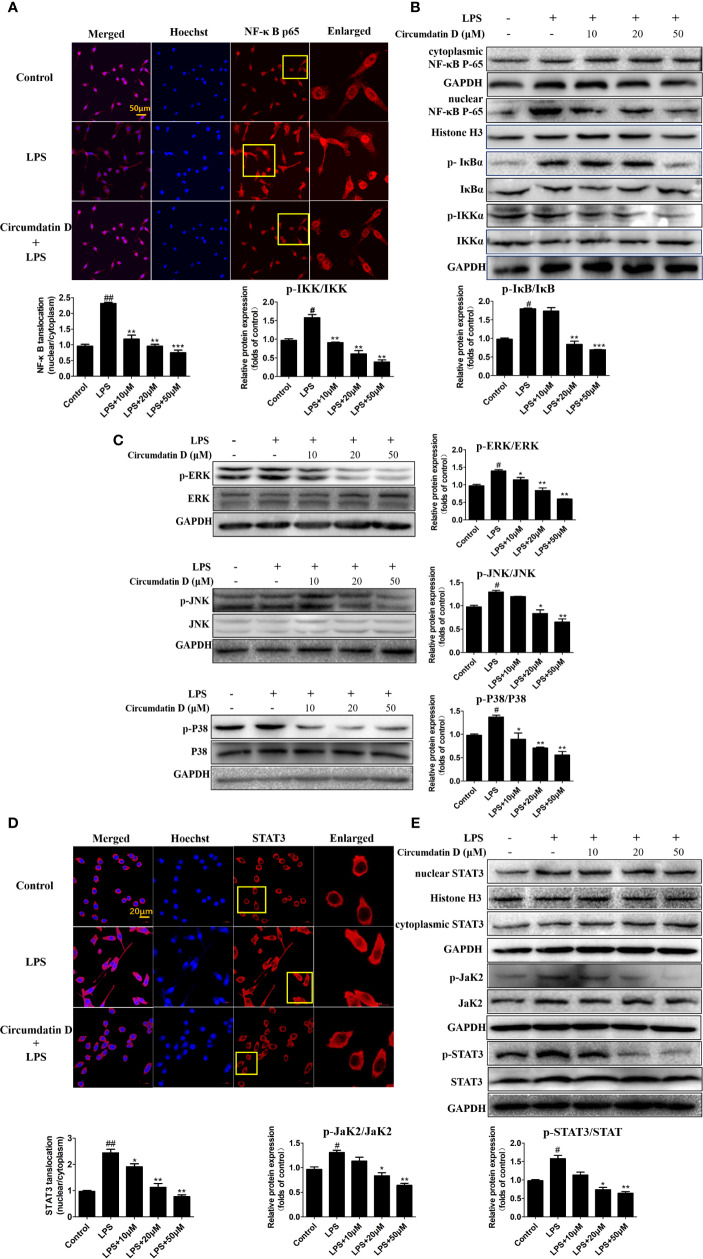
Circumdatin D inhibition of NF-κB/STAT3 activation and MAPK phosphorylation in LPS-induced BV-2 cells. **(A)** BV-2 cells were stimulated with LPS (1 μg/ml) in the absence or presence of circumdatin D (10, 20 and 50 μM) for 3 h, followed by detection of the NF-κB p65 subunit translocation by immunocytochemistry. Red fluorescence represents the NF-κB p65 subunit, and blue fluorescence represents nuclear Hoechst staining. **(B)** BV-2 cells were stimulated with LPS (1 μg/ml) in the absence or presence of circumdatin D (10, 20 and 50 μM) for 3 h, NF-κB p65 levels in the nucleus and cytoplasm, the phosphorylated and total IKKβ and IκB proteins were determined by Western blot. Histone H3 and GAPDH were used as endogenous controls for nuclear and cytoplasmic proteins, respectively. **(C)** BV-2 cells were stimulated with LPS (1 μg/ml) in the absence or presence of circumdatin D (10, 20 and 50 μM) for 3 h, the phosphorylated and total ERK1/2-JNK1/2-p38 MAPKs were determined by Western blot. **(D)** BV-2 cells were treated with LPS (1 μg/ml) in the absence or presence of circumdatin D (10, 20 and 50 μM) for 3 h, followed by detection of the STAT3 translocation by immunocytochemistry. Red fluorescence represents the STAT3, and blue fluorescence represents nuclear Hoechst staining. **(E)** STAT3 levels in the nucleus and cytoplasm, the phosphorylated and total JaK2 and STAT3 proteins were determined by Western blot. Histone H3 and GAPDH were used as endogenous controls for nuclear and cytoplasmic proteins, respectively. Values represent the mean ± SD of three independent experiments (^#^ compared with the control, *compared with LPS, *^/#^ P < 0.05, **^/##^ P < 0.01, ***P < 0.001).

In BV-2 microglial cells, circumdatin D inhibited LPS-induced NF-κB nuclear translocation as detected by immunofluorescence staining (**Figure 7A**). This result was further confirmed by western blot analysis of the expression of NF-κB p65 in the nucleus and in the cytoplasm. Nuclear accumulation of NF-κB p65 was increased in LPS-treated BV-2 cells as compared to control, whereas circumdatin D reduced nuclear NF-κB p65 levels significantly. This fact provided an evidence that circumdatin D inhibited LPS-induced NF-κB nuclear translocation. In addition, circumdatin D also down-regulated the phosphorylation levels of IKK and IκB, the important upstream signals of NF-κB dose-dependently (**Figure 7B**). Therefore, circumdatin D exerted inhibition of pro-inflammatory response in LPS-stimulated BV-2 cells *via* inhibiting the activation of NF-κB signaling pathway.

LPS rapidly activated the phosphorylation of mitogen-activated protein kinase (MAPK) signals, whereas western blot revealed that circumdatin D markedly suppressed the phosphorylation in LPS-induced BV-2 cells (**Figure 7C**). The result suggested that circumdatin D exerted its inhibition of pro-inflammatory response by downregulating MAPK signaling pathways.

**Figure 8:**
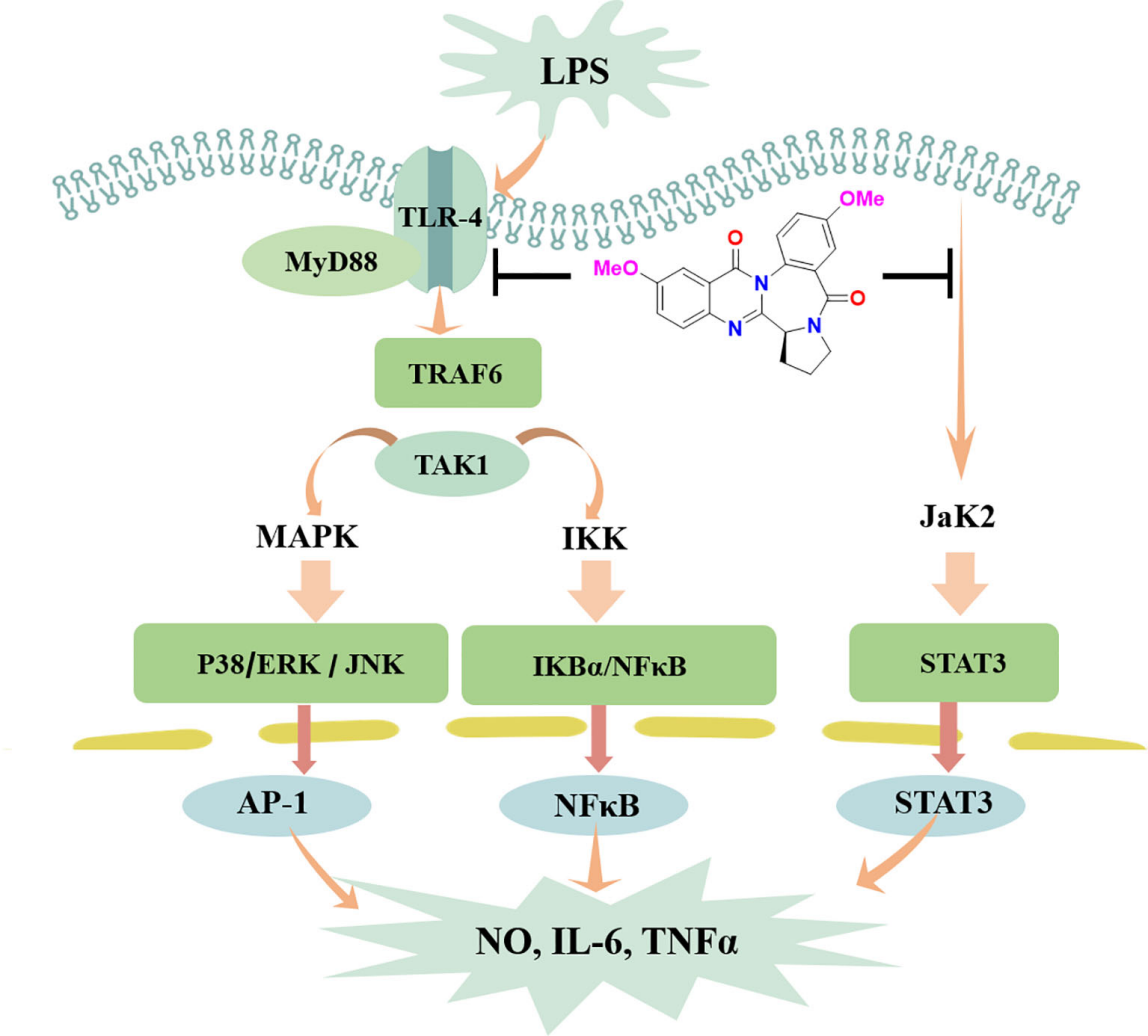
Schematic illustration of anti-inflammatory responses of circumdatin D. Circumdatin D negatively regulated TLR4-mediated NF-κB, MAPKs and JAK/STAT inflammatory signaling pathway and reduced the production of pro-inflammatory factors from LPS stimulated microglia. ERK, extracellular signal‐regulated kinase; JNK, c‐Jun N‐terminal kinase; NF‐kB, nuclear factor kappa‐light‐chain‐enhancer of activated B cells; TNF‐α, tumor necrosis factor‐α; IL‐1β, interleukin‐1β.

Activation of the STAT3 signaling pathway mediates inflammatory-associated factor expression (Zeng et al., 2012; Zeng et al., 2015). Herein, the translocation of STAT3 in response to LPS-induced neuroinflammation in BV-2 cells was examined. In LPS-treated BV-2 cells, circumdatin D effectively inhibited the phosphorylation and translocation of STAT3 from the cytoplasm to the nucleus. Moreover, circumdatin D strongly reduced LPS-induced phosphorylation of JaK2. In immunofluorescence staining experiment, circumdatin D inhibiting nuclear translocation of STAT3 was observed by the staining nucleus of LPS-activated BV-2 cells (**Figures 7D, E**). These results suggest that JaK2/STAT3 signaling pathway may be another potential anti-inflammatory mechanism of circumdatin D in BV-2 cells. Taken together, the proposed mechanism underlying the inhibition of pro-inflammatory response by circumdatin D in LPS-activated microglia *via* modulating TLR4-mediated NF-κB, MAPKs and JAK/STAT inflammatory pathways (**Figure 8**).

## Discussion

Present work provided a group of marine-derived alkaloids, circumdatins as a new natural scaffold with AChE and pro-inflammatory response dual inhibitory activity. Circumdatin D as the most active compound exhibited potential neuroprotective effects against microglial activation-mediated neurotoxicity. Mode of action investigation revealed that circumdatin D inhibited the TLR4-mediated NF-κB, MAPKs and JAK/STAT inflammatory signaling pathways.

Overactivated microglia are associated with many kinds of neurodegenerative disease through release of NO and various proinﬂammatory cytokines. (Tuppo and Arias, 2005; Cai et al., 2014; Shabab et al., 2017). LPS, a component derived from Gram-negative bacterial cell wall, can activate microglial cells to release inflammatory cytokines. Thus, LPS has been widely used for inducing pro-inflammatory response caused by microglial activation. Circumdatin D significantly inhibited LPS-induced NO production, as well as iNOS and COX-2 expression in BV-2 cells. In addition, circumdatin D significantly decreased TNF-α and IL-1β releasing in LPS-activated BV-2 cells, while those inflammatory cytokines augmented neurodegeneration and neuronal death. The attenuation of pro-inflammatory response by circumdatin D was also declared in primary microglia by decreasing LPS induced NO, TNF-α, and IL-1β production. In the inflammatory cascades, LPS is recognized by TLR4 on the surface of microglia to recruit and interact with MyD88, leading to the activation of down-stream MAPK and NF-κB signals, which subsequently regulated the release of pro-inflammatory cytokines (Wang et al., 2014; Rahimifard et al., 2017; Shabab et al., 2017). Thus, activation of TLR4 was the primary event in the induction of inflammatory processes. Circumdatin D attenuated LPS-induced TLR4 and MyD88 expression in BV-2 cells, indicating its attenuation of pro-inflammatory response initially induced by inhibiting the TLR4 signaling pathway. NF-κB plays an important role in the inflammatory responses and the release of IL-1β, TNF-α. In the active state, NF-κB liberates from IκBα through phosphorylation and degradation, and then translocates into the nucleus where it transcriptionally regulates proinflammatory genes expression. In the present study, circumdatin D attenuated NF-κB p65 subunit nuclear translocation and markedly decreased the IKKα phosphorylation and IκB degradation. These findings supported that circumdatin D exerted an effective anti-inflammatory activity *via* blockage TLR4-mediated NF-kB signaling pathway. The MAPK signaling pathway, ERK1/2-JNK1/2-p38 MAPKs, is also involved in LPS-induced inflammatory mediator expression and pro-inflammatory cytokine production (Yang et al., 2014; Cho et al., 2016; Shabab et al., 2017). These events agreed our experimental data that LPS increased the expression of phosphorylated ERK, p38 and JNK in BV-2 cells. As we expected, pretreatment of LPS-stimulated BV-2 microglia with circumdatin D significantly inhibited the phosphorylation of ERK, p38 and JNK. These findings concluded that circumdatin D interfered LPS-induced pro-inflammatory responses by suppressing activation of TLR4-dependent NF-κB and MAPKs signal pathways in BV-2 microglial cells. Multiple signal transduction pathways are involved in the induction of inflammatory factor production. JAK/STAT signaling pathway was explored to extend our understanding of inhibition of pro-inflammatory mechanism of circumdatin D. Herein, circumdatin D markedly inhibited phosphorylation of Jak2 as well as STAT3 nuclear translocation in LPS-activated BV-2 cells, suggesting that JAK/STAT signaling pathway is another potential mode of action for circumdatin D exerting inhibitory effects upon LPS-induced pro-inflammatory responses in BV-2 cells.

Prolonged activation of microglia resulted in a state of neurotoxicity in which overproduction of proinflammatory cytokines are harmful for neurons (Ransohoff, 2016; Hickman et al., 2018). Thus, inhibiting an uncontrolled neuro-inflammatory response in microglial cells may potentially prevent neuronal cells from damage. Based on circumdatin D obviously inhibiting LPS-induced production of pro-inflammatory factors, whether circumdatin D protected neurons against inflammation-induced neuronal death was conducted. In microglial-neuronal co-cultures, circumdatin D was found to protect neurons from inflammation-associated injury according to the data of neuronal viability, apoptosis and morphological assays. The results demonstrated that circumdatin D exhibited indirectly neuroprotective effects against microglia activation-mediated neurotoxicity induced by LPS. Cholinergic deficiency is an important cause of AD, and AChE is still the most viable therapeutic target for AD therapy (Beach et al., 2000; Ferreira-Vieira et al., 2016). Circumdatin D showed inhibitory effects toward AChE, that was corresponded with the strong interaction between circumdatin D and AChE in SPR experiment. Furthermore, circumdatin D suppressed AChE activity in LPS-stimulated rat primary neuronal cells and increased cell viability, thus alleviated the corresponding apoptosis. These findings were relevant to the reported results that cell apoptosis associated with AChE expression (Zhang et al., 2002; Pérez-Aguilar et al., 2015), suggesting the neuroprotective effects of circumdatin D partly related to downregulating AChE expression.

*C. elegans* strain CL4176 expresses Aβ in muscle cells upon temperature up-shift, leading to progressive neurodegeneration and paralysis, and thus has been employed as a valid AD model. In addition, the pathological features of CL4176 include AChE and inflammation gene overexpression (Link et al., 2003; Link, 2006; Xin et al., 2013; Cacabelos et al., 2014; Li et al., 2018). In present work, CL4176 was used as an AD pathological model to evaluate the efficacy of circumdatin D *in vivo*. Bioassay data indicated that circumdatin D effectively reduced the paralysis of CL4176 nematodes upon temperature up-shift comparing to untreated animals. In addition, circumdatin D significantly inhibited the AChE activities and relevant gene expression in *C. elegans* C4176. Acetylcholine has been reported to be intimately associated with *C. elegans* behaviors such as muscle contraction, paralysis, and locomotion by affecting interneuronal, neuromuscular, and neuroendothelial signaling (Ahmad and Ebert, 2017). Therefore, the reduced paralysis by circumdatin D in the CL4176 nematodes could be attributed to its AChE inhibiting activities, which could prevent the breakdown of acetylcholine and maintains its normal levels. F22E5.6 and ZC239.12 genes are closely related to inflammation response in CL4176 nematodes (Link et al., 2003; Link, 2006; Xin et al., 2013). Circumdatin D significantly reduced these inflammation genes expression in CL4176 worms induced by temperature up-shift. Thus, circumdatin D effectively improved the paralysis of CL4176 nematodes *via* suppression of AChE activity and inflammatory-related gene expression in the *in vivo* test.

In summary, present study revealed that circumdatin D exerted neuroprotective effects by attenuating pro-inflammatory response and downregulating acetylcholinesterase activity *in vitro* and *in vivo*. Although further studies are required to test circumdatin D in other animal models, current data suggested that circumdatin D may be a reliable and promising multifunctional drug candidate for treating AD.

## Data Availability Statement

The raw data supporting the conclusions of this article will be made available by the authors, without undue reservation, to any qualified researcher.

## Author Contributions

CZ was mainly responsible for designing experiments and conducting related research and subsequent data analysis, and finally completed the writing of the manuscript. LH is responsible for the separation of related compounds circumdatins. DL provides technical assistance through several protocols. JH and WL conceived the study as a whole and coordinated the entire project. The final draft was read and endorsed by all authors.

## Funding

This work was supported by National Key Research and Development Program of China (No. YFC0310900), NSFC (81991525, 21861142006, 81872793, 81630089), COMRA DY135-B-05 and 2018ZX09711001-001-008.

## Conflict of Interest

The authors declare that the research was conducted in the absence of any commercial or financial relationships that could be construed as a potential conflict of interest.

## References

[B1] Ahmad W. Ebert P. R. (2017). Metformin attenuates Aβ pathology mediated through levamisole sensitive nicotinic acetylcholine receptors in a C. elegans model of Alzheimer's disease. Mol. Neurobiol. 54, 5427–5439. 10.1007/s12035-016-0085-y 27596506

[B2] Anand P. Singh B. (2013). A review on cholinesterase inhibitors for Alzheimer's disease. Arch. Pharm. Res. 36, 375–399. 10.1007/s12272-013-0036-3 23435942

[B3] Beach T. G. Kuo Y. M. Spiegel K. Emmerling M. R. Sue L. I. Kokjohn K. (2000). The cholinergic deficit coincides with Abeta deposition at the earliest histopathologic stages of Alzheimer disease. J. Neuropathol. Exp. Neurol. 59, 308–313. 10.1093/jnen/59.4.308 10759186

[B4] Behrens S. Rattinger G. B. Schwartz S. Matyi J. Sanders C. DeBerard M. S. (2018). Use of FDA approved medications for Alzheimer's disease in mild dementia is associated with reduced informal costs of care. Int. Psychogeriatr. 30, 1499–1507. 10.1017/S104161021800011X 29559029 PMC6150839

[B5] Bock M. G. Dipardo R. M. Rittle K. E. Evans B. E. Freidinger R. M. Veber D. F. (1986). Cholecystokinin antagonists. synthesis of asperlicin analogs with improved potency and water solubility. J. Med. Chem. 29, 1941–1945. 10.1002/chin.198711211 3761313

[B6] Briggs R. Kennelly S. P. O'Neill D. (2016). Drug treatments in Alzheimer's disease. Clin. Med. (Lond) 16, 247–253. 10.7861/clinmedicine 27251914 PMC5922703

[B7] Cacabelos R. Cacabelos P. Torrellas C. Tellado I. Carril J. C. (2014). Pharmacogenomics in Drug Discovery and Development. Pharmacogenomics of Alzheimer"s disease: Novel therapeutic strategies for drug development (New York, NY: Humana Press), 323–556. 10.1007/978-1-4939-0956-8_13 25150875

[B8] Cai Z. Hussain M. D. Yan L. J. (2014). Microglia, neuroinflammation, and beta-amyloid protein in Alzheimer's disease. Int. J. Neurosci. 124, 307–321. 10.3109/00207454.2013.833510 23930978

[B9] Cho K. H. Kim D. C. Yoon C. S. Ko W. M. Lee S. J. Sohn J. H. (2016). Anti-neuroinflammatory effects of citreohybridonol involving TLR4-MyD88-mediated inhibition of NF-кB and MAPK signaling pathways in lipopolysaccharide-stimulated BV2 cells. Neurochem. Int. 95, 55–62. 10.1016/j.neuint.2015.12.010 26724567

[B10] Chuan M. C. Xiao M. L. Chun S. L. Hao F. S. Shu S. G. Bin G. W. (2009). Benzodiazepine alkaloids from marine-derived endophytic fungus *Aspergillus Ochraceus*. Helv. Chim. Acta 92, 1366–1370. 10.1002/hlca.200900084

[B11] Colonna M. Butovsky O. (2017). Microglia function in the central nervous system during health and neurodegeneration. Annu. Rev. Immunol. 35, 441–468. 10.1146/annurev-immunol-051116-052358 28226226 PMC8167938

[B12] Cowan M. Petri W. A. Jr. (2018). Microglia: immune regulators of neurodevelopment. Front. Immunol. 9, 2576. 10.3389/fimmu.2018.02576 30464763 PMC6234957

[B13] Cummings J. Lee G. Ritter A. Sabbagh M. Zhong K. (2019). Alzheimer's disease drug development pipeline: 2019. Alzheimers Dement. (N. Y.) 5, 272–293. 10.1016/j.trci.2019.05.008 31334330 PMC6617248

[B14] de Freitas Silva M. Dias K. S. T. Gontijo V. S. Ortiz C. J. C. Viegas C. Jr. (2018). Multi-Target Directed Drugs as a Modern Approach for Drug Design Towards Alzheimer's Disease: An Update. Curr. Med. Chem. 25, 3491–3525. 10.2174/0929867325666180111101843 29332563

[B15] Dong Y. Li X. Cheng J. Hou L. (2019). Drug development for Alzheimer's disease: microglia induced neuroinflammation as a target? Int. J. Mol. Sci. 20, E558. 10.3390/ijms20030558 30696107 PMC6386861

[B16] Ferreira-Vieira T. H. Guimaraes I. M. Silva F. R. Ribeiro F. M. (2016). Alzheimer's disease: Targeting the cholinergic system. Curr. Neuropharmacol. 14, 101–115. 10.2174/1570159x13666150716165726 26813123 PMC4787279

[B17] Galimberti D. Scarpini E. (2016). Old and new acetylcholinesterase inhibitors for Alzheimer's disease. Expert. Opin. Investig. Drugs 25, 1181–1187. 10.1080/13543784.2016.1216972 27459153

[B18] Hansen D. V. Hanson J. E. Sheng M. (2018). Microglia in Alzheimer's disease. J. Cell. Biol. 217, 459–472. 10.1083/jcb.201709069 29196460 PMC5800817

[B19] Hardy J. Selkoe D. J. (2002). The amyloid hypothesis of Alzheimer's disease: progress and problems on the road to therapeutics. Science 297, 35335–35336. 10.1126/science.1072994 12130773

[B20] Heneka M. T. Kummer M. P. (2014). Innate immune activation in neurodegenerative disease. Nat. Rev. Immunol. 14, 463–477. 10.1038/nri3705 24962261

[B21] Hickman S. Izzy S. Sen P. Morsett L. El Khoury J. (2018). Microglia in neurodegeneration. Nat. Neurosci. 21, 1359–1369. 10.1038/s41593-018-0242-x 30258234 PMC6817969

[B22] Hu L. Zhang T. Liu D. Guan G. Huang J. Proksch P. (2019). Notoamide-type alkaloid induced apoptosis and autophagy via a P38/JNK signaling pathway in hepatocellular carcinoma cells. RSC Adv. 9, 19855–19868. 10.1039/C9RA03640G PMC9065365 35519412

[B23] Hung S. Y. Fu W. M. (2017). Drug candidates in clinical trials for Alzheimer's disease. J. Biomed. Sci. 24, 47. 10.1186/s12929-017-0355-7 28720101 PMC5516350

[B24] Khoury R. Patel K. Gold J. Hinds S. Grossberg G. T. (2017). Recent Progress in the Pharmacotherapy of Alzheimer's Disease. Drugs Aging 34, 811–820. 10.1007/s40266-017-0499-x 29116600

[B25] Kumar A. Singh A. (2015). A review on Alzheimer's disease pathophysiology and its management: an update. Pharmacol. Rep. 67, 195–203. 10.1016/j.pharep.2014.09.004 25712639

[B26] Li X. Wang H. Lu Z. Zheng X. Ni W. Zhu J. (2016). Development of multifunctional pyrimidinylthiourea derivatives as potential anti-Alzheimer agents. J. Med. Chem. 59, 8326–8344. 10.1021/acs.jmedchem.6b00636 27552582

[B27] Li Y. Guan S. Liu C. Chen X. Zhu Y. Xie Y. (2018). Neuroprotective effects of Coptis chinensis Franch polysaccharide on amyloid-beta (Aβ)-induced toxicity in a transgenic Caenorhabditis elegans model of Alzheimer's disease (AD). Int. J. Biol. Macromol. 113, 991–995. 10.1016/j.ijbiomac.2018.03.035 29524490

[B28] Link C. D. Taft A. Kapulkin V. Duke K. Kim S. Fei Q. (2003). Gene expression analysis in a transgenic Caenorhabditis elegans Alzheimer's disease model. Neurobiol. Aging 24, 397–413. 10.1016/s0197-4580(02)00224-5 12600716

[B29] Link C. D. (2006). C. elegans models of age-associated neurodegenerative diseases: lessons from transgenic worm models of Alzheimer's disease. Exp. Gerontol. 41, 1007–1013. 10.1016/j.exger.2006.06.059 16930903

[B30] Liu C. Y. Wang X. Liu C. Zhang H. L. (2019). Pharmacological targeting of microglial activation: New therapeutic approach. Front. Cell. Neurosci. 13, 514. 10.3389/fncel.2019.00514 31803024 PMC6877505

[B31] López-Gresa M. P. González M. C. Primo J. Moya P. Romero V. Estornell E. (2005). Circumdatin H, a new inhibitor of mitochondrial NADH oxidase, from *Aspergillus Ochraceus*. J. Antibiot. 58, 416–419. 10.1002/chin.200551178 16156520

[B32] Lublin A. L. Link C. D. (2013). Alzheimer's disease drug discovery: in vivo screening using Caenorhabditis elegans as a model for β-amyloid peptide-induced toxicity. Drug Discovery Today Technol. 10, e115–e119. 10.1016/j.ddtec.2012.02.002 PMC3640579 24050239

[B33] Mesiti F. Chavarria D. Gaspar A. Alcaro S. Borges F. (2019). The chemistry toolbox of multitarget-directed ligands for Alzheimer's disease. Eur. J. Med. Chem. 181, 111572. 10.1016/j.ejmech 31404859

[B34] Ng Y. P. Or T. C. Ip N. Y. (2015). Plant alkaloids as drug leads for Alzheimer's disease. Neurochem. Int. 89, 260–270. 10.1016/j.neuint.2015.07.018 26220901

[B35] Ni M. Aschner M. (2010). Neonatal rat primary microglia: isolation, culturing, and selected applications. Curr. Protoc. Toxicol. 43, 12.17.1–12.17.16. 10.1002/0471140856.tx1217s43. Chapter 12, Unit 12.17. PMC2959194 20960423

[B36] Pérez-Aguilar B. Vidal C. J. Palomec G. García-Dolores F. Gutiérrez-Ruiz M. C. Bucio L. (2015). Acetylcholinesterase is associated with a decrease in cell proliferation of hepatocellular carcinoma cells. Biochim. Biophys. Acta 1852, 1380–1387. 10.1016/j.bbadis.2015.04.003 25869328

[B37] Rahimifard M. Maqbool F. Moeini-Nodeh S. Niaz K. Abdollahi M. Braidy N. (2017). Targeting the TLR4 signaling pathway by polyphenols: A novel therapeutic strategy for neuroinflammation. Ageing Res. Rev. 36, 11–19. 10.1016/j.arr.2017.02.004 28235660

[B38] Ransohoff R. M. (2016). How neuroinflammation contributes to neurodegeneration. Science 353, 777–783. 10.1126/science.aag2590 27540165

[B39] Ritchie C. W. Ames D. Clayton T. Lai R. (2004). Metaanalysis of randomized trials of the efficacy and safety of donepezil, galantamine, and rivastigmine for the treatment of Alzheimer disease. Am. J. Geriatr. Psychiatry 12, 358–369. 10.1176/appi.ajgp.12.4.358 15249273

[B40] Salter M. W. Stevens B. (2017). Microglia emerge as central players in brain disease. Nat. Med. 23, 1018–1027. 10.1038/nm.4397 28886007

[B41] Schain M. Kreisl W. C. (2017). Neuroinflammation in neurodegenerative disorders-a review. Curr. Neurol. Neurosci. Rep. 7, 25. 10.1007/s11910-017-0733-2 28283959

[B42] Shabab T. Khanabdali R. Moghadamtousi S. Z. Kadir H. A. Mohan G. (2017). Neuroinflammation pathways: a general review. Int. J. Neurosci. 127, 624–633. 10.1080/00207454.2016.1212854 27412492

[B43] Shih R. H. Wang C. Y. Yang C. M. (2015). NF-kappaB Signaling Pathways in Neurological Inflammation: A Mini Review. Front. Mol. Neurosci. 8, 77. 10.3389/fnmol.2015.00077 26733801 PMC4683208

[B44] Sun H. H. Barrow C. J. Sedlock D. M. Gillum A. M. Cooper R. (1994). Benzomalvins, new suhstance P inhibitors from a *penicillium sp*. J. Antibiot. 47, 515–522. 10.7164/antibiotics.47.515 7518818

[B45] Sun W. Chen L. Zheng W. Wei X. Wu W. Duysen E. G. (2017). Study of acetylcholinesterase activity and apoptosis in SH-SY5Y cells and mice exposed to ethanol. Toxicology 384, 33–39. 10.1016/j.tox.2017.04.007 28427893

[B46] Szwajgier D. (2015). Anticholinesterase activity of selected phenolic acids and flavonoids - interaction testing in model solutions. Ann. Agric. Environ. Med. 22, 690–694. 10.5604/12321966.1185777 26706979

[B47] Tuppo E. E. Arias H. R. (2005). The role of inflammation in Alzheimer's disease. Int. J. Biochem. Cell. Biol. 37, 289–305. 10.1016/j.biocel.2004.07.009 15474976

[B48] Wang X. Wang C. Wang J. Zhao S. Zhang K. Wang J. (2014). Pseudoginsenoside-F11 (PF11) exerts anti-neuroinflammatory effects on LPS-activated microglial cells by inhibiting TLR4-mediated TAK1/IKK/NF-κB, MAPKs and Akt signaling pathways. Neuropharmacology 79, 642–656. 10.1016/j.neuropharm.2014.01.022 24467851

[B49] Wang T. Liu X. H. Guan J. Ge S. Wu M. B. Lin J. P. (2019). Advancement of multi-target drug discoveries and promising applications in the field of Alzheimer's disease. Eur. J. Med. Chem. 169, 200–223. 10.1016/j.ejmech.2019.02.076 30884327

[B50] Xin L. Yamujala R. Wang Y. Wang H. Wu W. H. Lawton M. A. (2013). Acetylcholineestarase-inhibiting alkaloids from Lycoris radiata delay paralysis of amyloid beta-expressing transgenic C. elegans CL4176. PloS One 8, e63874. 10.1371/journal.pone.0063874 23675513 PMC3652842

[B51] Yang J. M. Rui B. B. Chen C. Chen H. Xu T. J. Xu W. P. (2014). Acetylsalicylic acid enhances the anti-inflammatory effect of fluoxetine through inhibition of NF-κB, p38-MAPK and ERK1/2 activation in lipopolysaccharide-induced BV-2 microglia cells. Neuroscience 275, 296–304. 10.1016/j.neuroscience.2014.06.016 24952332

[B52] Zeng K. W. Zhang T. Fu H. Liu G. X. Wang X. M. (2012). Schisandrin B exerts anti-neuroinflammatory activity by inhibiting the Toll-like receptor 4-dependent MyD88/IKK/NF-κB signaling pathway in lipopolysaccharide-induced microglia. Eur. J. Pharmacol. 692, 29–37. 10.1016/j.ejphar.2012.05.030 22698579

[B53] Zeng K. W. Yu Q. Liao L. X. Song F. J. Lv H. N. Jiang Y. (2015). Anti-Neuroinflammatory Effect of MC13, a Novel Coumarin Compound From Condiment Murraya, Through Inhibiting Lipopolysaccharide-Induced TRAF6-TAK1-NF-κB, P38/ERK MAPKS and Jak2-Stat1/Stat3 Pathways. J. Cell. Biochem. 116, 1286–1299. 10.1002/jcb.25084 25676331

[B54] Zhang X. J. Yang L. Zhao Q. Caen J. P. He H. Y. Jin Q. H. (2002). Induction of acetylcholinesterase expression during apoptosis in various cell types. Cell Death. Differ. 9, 790–800. 10.1038/sj.cdd.4401034 12107822

