# Supplementary material for: Circumdatin D Exerts Neuroprotective Effects by Attenuating LPS-Induced Pro-Inflammatory Responses and Downregulating Acetylcholinesterase Activity In Vitro and In Vivo
Source: Front Pharmacol. 2020 May 25;11:760. doi: 10.3389/fphar.2020.00760 (PMC7261837; doi:10.3389/fphar.2020.00760)
Supplement: Supplementary file 1 [file DataSheet_1.docx]

**Circumdatin D Exerts Neuroprotective Effects by Attenuating LPS-induced Pro-inflammatory Responses and Downregulating Acetylcholinesterase Activity *in vitro* and *in vivo***

**Supplemental Material**

1. **Supplementary Methods**
2. **Spectroscopic data of the isolated compounds**

Circumdatin F (**1**), pale solid, [*α*]^25^_D_ -151.0 (*c* 0.1, MeOH). UV (MeOH) λmax 254 nm; ^1^H-NMR (DMSO-*d*_6_, 400 MHz) *δ*_H_ 8.81 (1H, d, *J* = 4.0 Hz, NH), 7.79 (1H, d, *J* = 7.5 Hz, H-3), 7.64 (1H, t, *J* = 7.5 Hz, H-4), 7.73 (1H, t, *J* = 7.5 Hz, H-5), 7.60 (1H, d, *J* = 7.5 Hz, H-6), 8.19 (1H, d, *J* = 7.8 Hz, H-10), 7.62 (1H, t, *J* = 7.8 Hz, H-11), 7.90 (1H, t, *J* = 7.8 Hz, H-12), 7.76 (1H, d, *J* = 7.8 Hz, H-13), 4.43 (1H, q, *J* = 6.5 Hz, H-16), 1.55 (3H, d, *J* = 6.5 Hz, H-17); ^13^C-NMR (100 MHz, DMSO-*d*6) *δ*_C_ 167.1 (C-1), 131.8 (C-2), 129.3 (C-3), 129.2 (C-4), 127.9 (C-5), 131.1 (C-6), 133.5 (C-7), 161.5 (C-8), 121.5 (C-9), 127.3 (C-10), 129.1 (C-11), 135.6 (C-12), 128.0 (C-13), 146.4 (C-14), 157.0 (C-15), 50.0 (C-16), 15.3 (C-17); ESIMS m/z 292 [M+H]^+^. The spectroscopic data were comparable to those reported for circumdatin F (Rahbæk et al., 1999).

Circumdatin C (**2**), yellow solid, [*α*]^25^_D_ -152.0 (*c* 0.1, MeOH). UV (MeOH) λmax 254 nm; ^1^H-NMR (DMSO-*d*_6_, 400MHz) *δ*_H_ 10.20（1H，s，OH), 8.70 (1H，d，*J* = 5.7 Hz, NH), 7.12 (1H, brd, *J* = 2.6 Hz, H-3), 7.02 (1H, dd, *J* = 2.6, 8.8 Hz, H-5), 7.42 (1H, d, *J* = 8.8 Hz, H-6), 8.16 (1H, d, *J* = 7.7 Hz, H-10), 7.61 (1H, dd, *J* = 7.4, 7.7 Hz, H-11), 7.92 (1H, dd, *J* = 7.4, 8.1 Hz, H-12), 7.71 (1H, d, *J* = 8.1 Hz, H-13), 4.42 (1H, dq, *J* = 5.7, 6.6 H-16), 1.52 (3H, d, *J* = 6.6 Hz, H-17). ^13^C-NMR (100MHz, DMSO-*d*6) *δ*_C_ 167.1 (C-1), 132.8 (C-2), 114.6 (C-3), 157.7 (C-4), 118.3 (C-5), 130.4 (C-6), 124.8 (C-7), 161.6 (C-8), 121.4 (C-9), 127.2 (C-10), 127.8 (C-11), 135.4 (C-12), 127.8 (C-13), 146.4 (C-14), 157.3 (C-15), 50.0 (C-16), 15.3 (C-17). ESIMS *m/z* 308 [M+H]^+^. The spectroscopic data were comparable to those reported for circumdatin C (Rahbæk et al., 1999).

Circumdatin G (**3**), pale-yellow solid, [*α*]^25^_D_ -244.0 (*c* 0.1, MeOH). UV (MeOH) λmax 254 nm; ^1^H-NMR (DMSO-*d*_6_, 400MHz) *δ*_H_ 8.79 (1H, d, *J* = 5.5 Hz, NH), 7.78 (1H, d, *J* = 6.9 Hz, H-3), 7.58 (1H, t, *J* = 6.9 Hz, H-4), 7.60 (1H, dd, *J* = 6.9, 7.6 Hz, H-5), 7.71 (1H, d, *J* = 7.6 Hz, H-6), 7.60 (1H, d, *J* = 8.0 Hz, H-10), 7.42 (1H, t, *J* = 8.0, Hz, H-11), 7.31 (1H, d, *J* = 8.0 Hz, H-12), 4.31 (1H, dq, *J* = 5.5, 6.4 Hz, H-16), 1.58 (3H, d, *J* = 6.4 Hz, H-17); ^13^C-NMR (100 MHz, DMSO-*d*6) *δ*_C_ 167.2 (C-1), 131.8 (C-2), 129.3 (C-3), 129.2 (C-4), 131.2 (C-5), 119.8 (C-6), 133.7 (C-7), 161.6 (C-8), 122.4 (C-9), 129.2 (C-10), 128.5 (C-11), 117.0 (C-12), 153.5 (C-13), 135.2 (C-14), 155.3 (C-15), 50.1 (C-16), 15.4 (C-17), ESIMS *m/z* 308 [M+H]^+^. The spectroscopic data were comparable to those reported for circumdatin G (Dai et al., 2001).

Circumdatin I (**4**), pale-yellow solid, [*α*]^25^_D_ -214.0 (*c* 0.1, MeOH). UV (MeOH) λmax 254 nm; ^1^H-NMR (DMSO-*d*_6_, 400 MHz) *δ*_H_ 10.17（1H, s, OH), 9.68 (1H, s, OH), 8.67 (1H, d, *J* = 5.6 Hz, NH), 7.10 (1H, brd, *J* = 2.8 Hz, H-3), 7.02 (1H, dd, *J* = 2.8, 8.8 Hz, H-5), 7.60 (1H, d, *J* = 8.8 Hz, H-6), 7.60 (1H, d, *J* = 8.0 Hz, H-10), 7.39 (1H, dd, *J* = 7.6, 8.0 Hz, H-11), 7.28 (1H, d, *J* = 7.6 Hz, H-12), 4.32 (1H, m, H-16), 1.57 (3H, d, *J* = 6.4 Hz, H-17); ^13^C-NMR (100 MHz, DMSO-*d*6) *δ*_C_ 167.1 (C-1), 132.9 (C-2), 114.3 (C-3), 157.7 (C-4), 117.0 (C-5), 130.5 (C-6), 133.7 (C-7), 161.7 (C-8), 122.4 (C-9), 129.2 (C-10), 128.5 (C-11), 119.8 (C-12), 153.4 (C-13), 135.2 (C-14), 155.6 (C-15), 50.0 (C-16), 15.4 (C-17); ESIMS *m/z* 324 [M+H]^+^. The spectroscopic data were comparable to those reported for circumdatin I (Zhang et al., 2008).

5-Hydroxycircumdatin C (**5**), white powder, [*α*]^25^_D_ -99.0 (*c* 0.1, MeOH). UV (MeOH) λmax 254 nm; ^1^H-NMR (DMSO-*d*_6_, 400 MHz) *δ*_H_ 8.48 (1H, d, *J* = 5.7 Hz, NH), 7.10 (1H, s, H-3), 6.91 (1H, s, H-6), 8.17 (1H, d, *J* = 7.8 Hz, H-10), 7.58 (1H, dd, *J* = 7.5, 7.8 Hz, H-11), 7.88 (1H, t, *J* = 7.5 Hz, H-12), 7.72 (1H, d, *J* = 7.5 Hz, H-13), 4.34 (1H, m, H-16), 1.51 (1H, d, J = 6.5 Hz, H-17); ^13^C-NMR (100 MHz, DMSO-*d*6) *δ*_C_ 167.3 (C-1), 123.0 (C-2), 115.0 (C-3), 146.4 (C-4), 147.8 (C-5), 115.8 (C-6), 125.6 (C-7), 161.5 (C-8), 121.5 (C-9), 127.2 (C-10), 127.8 (C-11), 135.4 (C-12), 127.8 (C-13), 146.2 (C-14), 157.5 (C-15), 49.9 (C-16), 15.4 (C-17), ESIMS *m/z* 324 [M+H]^+^. The spectroscopic data were comparable to those reported for 2-hydroxycircumdatin C (Cui et al., 2009).

Circumdatin H (**6**), white powder, [*α*]^25^_D_ -35.0 (*c* 0.1, MeOH). UV (MeOH) λmax 254 nm; ^1^H-NMR (DMSO-*d*_6_, 400 MHz) *δ*_H_ 7.55 (1H, d, *J* = 7.5 Hz, H-3), 7.48 (1H, t, *J* = 7.5 Hz, H-4), 7.40 (1H, t, *J* = 7.5 Hz, H-5), 7.92 (1H, d, *J* = 7.5 Hz, H-6), 7.48 (1H, d, *J* = 2.5 Hz, H-10), 7.21 (1H, dd, *J* = 2.5, 8.9 Hz, H-12), 7.53 (1H, d, *J* = 8.9 Hz, H-13), 4.65 (1H, dd, J = 2.2, 6.5 Hz, H-16), 2.12 (1H, m, H-17a), 2.91 (1H, m, H-17b), 1.98 (1H, m, H-18a), 2.12 (1H, m, H-18b), 3.45 (1H, m, H-19a), 3.60 (1H, m, H-19b), 3.88 (3H, s, MeO); ^13^C-NMR (100 MHz, DMSO-*d*6) *δ*_C_ 163.8 (C-1), 133.9 (C-2), 123.8 (C-3), 125.0 (C-4), 127.6 (C-5), 131.0 (C-6), 126.5 (C-7), 161.6 (C-8), 122.4 (C-9), 107.3 (C-10), 158.8 (C-11), 124.7 (C-12), 129.6 (C-13), 140.6 (C-14), 153.0 (C-15), 58.9 (C-16), 26.9 (C-17), 23.7 (C-18), 47.6 (C-19), 56.2 (MeO); ESIMS *m/z* 348 [M+H]^+^. The spectroscopic data were comparable to those reported for circumdatin H (López-Gresa et al., 2005),

Circumdatin J (**7**), white powder, [*α*]^25^_D_ -102.0 (*c* 0.1, MeOH). UV (MeOH) λmax 254 nm; ^1^H-NMR (DMSO-*d*_6_, 400 MHz) *δ*_H_ 7.28 (1H, brd, *J* = 2.6 Hz, H-3), 7.21 (1H, dd, *J* = 2.6, 8.9 Hz, H-5), 7.53 (1H, d, *J* = 8.9 Hz, H-6), 7.55 (1H, d, *J* = 2.5 Hz, H-10), 7.48 (1H, dd, *J* = 2.5, 8.8 Hz, H-12), 7.69 (1H, d, *J* = 8.8 Hz, H-13), 4.65 (1H, dd, *J* = 4.0, 6.5 Hz, H-16), 2.91 (1H, m, Ha-17), 2.12 (1H, m, Hb-17), 2.12 (1H, m, Ha-18), 1.98 (1H, m, Hb-18), 3.60 (1H, m, Ha-19), 3.45 (1H, m, Hb-19), 3.88 (3H, s, MeO), 3.89 (3H, s, MeO). ^13^C-NMR (100 MHz, DMSO-*d*6) *δ*_C_ 163.8 (C-1), 133.9 (C-2), 112.8 (C-3), 159.0 (C-4), 117.6 (C-5), 131.0 (C-6), 126.5 (C-7), 161.6 (C-8), 122.4 (C-9), 107.3 (C-10), 158.8 (C-11), 124.7 (C-12), 129.6 (C-13), 140.6 (C-14), 153.0 (C-15), 58.9 (C-16), 26.9 (C-17), 23.7 (C-18), 46.6 (C-19), 56.2 (MeO), 56.1 (MeO), ESIMS *m/z* 378 [M+H]^+^. The spectroscopic data were comparable to those reported for circumdatin J (Ookura et al., 2008).

**2. Cell Surviability Detection**

Cell surviability was detected using the MTT assay. Cells were seeded in 96-well plates and incubated overnight, following which the cells were treated with different concentrations of circumdatins with or without 1µg/mL of LPS for indicated time. Then, the cells were subsequently cultured with MTT and incubated for 4 hours at 37°C. Thereafter, the purple formazan dye was dissolved in DMSO and the absorbance was measured at 570 nm.

**3. Nematode Toxicity Assay**

To eliminate the confounding factor of toxicity in the procedure, CL4176 worms were exposed to increasing concentrations of circumdatin D dissolved in NGM (0, 100, 200, 500 µM) for 24 h and 48h at 16 °C, respectively. Further, nematodes were exposed to 0.4% DMSO as the negative control. The animals were scored as live or death using a Motic dissecting microscope (Motic Inc., LTD., Hong Kong, China). The worms were considered as death if there was no response when gently touched with a platinum wire. Each independent assay included three NGM plates with thirty worms in each one. Toxicity was expressed as a total percentage of worm survival.

**4. Paralysis Assay**

In paralysis assay, CL4176 worms were egg-synchronized onto fresh NGM plates containing OP50 bacteria at 16℃. To induce the transgene expression, the temperature raised from 16 to 25℃ and lasted until the end of the paralysis assay. The recording was performed at two hours interval typically after 24h at 25 ^o^C until the majority of worm became paralyzed. The worms were considered as paralyzed if there was no response or they only moved their head when gently touched with a platinum wire. Each independent assay included three NGM plates with twenty worms in each one.

**II. Supplementary Figures**

**Figure S1.** ^1^H-NMR spectrum of circumdatin F (**1**) (DMSO-*d*_6_, 400 MHz)

**Figure S2.** ^13^C-NMR spectrum of circumdatin F (**1**) (DMSO-*d*_6_, 100 MHz)

**Figure S3.** ^1^H-NMR spectrum of circumdatin C (**2**) (DMSO-*d*_6_, 400 MHz）

**Figure S4.** ^13^C-NMR spectrum of circumdatin C (**2**) (DMSO-*d*_6_, 100 MHz)

**Figure S5.** ^1^H-NMR spectrum of circumdatin G (**3**) (DMSO-*d*_6_, 400 MHz)

**Figure S6.** ^13^C-NMR spectrum of circumdatin G (**3**) (DMSO-*d*_6_, 100 MHz)

**Figure S7.** ^1^H-NMR spectrum of circumdatin I (**4**) (DMSO-*d*_6_, 400 MHz)

**Figure S8.** ^13^C-NMR spectrum of circumdatin I (**4**) (DMSO-*d*_6_, 100 MHz)

**Figure S9.** ^1^H-NMR spectrum of 5-hydroxycircumdatin C (**5**) (DMSO-*d*_6_, 400 MHz)

**Figure S10.** ^13^C-NMR spectrum of 5-hydroxycircumdatin C (**5**) (DMSO-*d*_6_, 100 MHz)

**Figure S11.** ^1^H-NMR spectrum of circumdatin H (**6**) (DMSO-*d*_6_, 400 MHz)

**Figure S12.** ^13^C-NMR spectrum of circumdatin H (**6**) (DMSO-*d*_6_, 100 MHz)

**Figure S13.** ^1^H-NMR spectrum of circumdatin J (**7**) (DMSO-*d*_6_, 400 MHz)

**Figure S14.** ^13^C-NMR spectrum of circumdatin J (**7**) (DMSO-*d*_6_, 100 MHz)

Detection wavelength: 210 nm

**Figure S15**. HPLC chromatograph of circumdatin J (**7**)

**
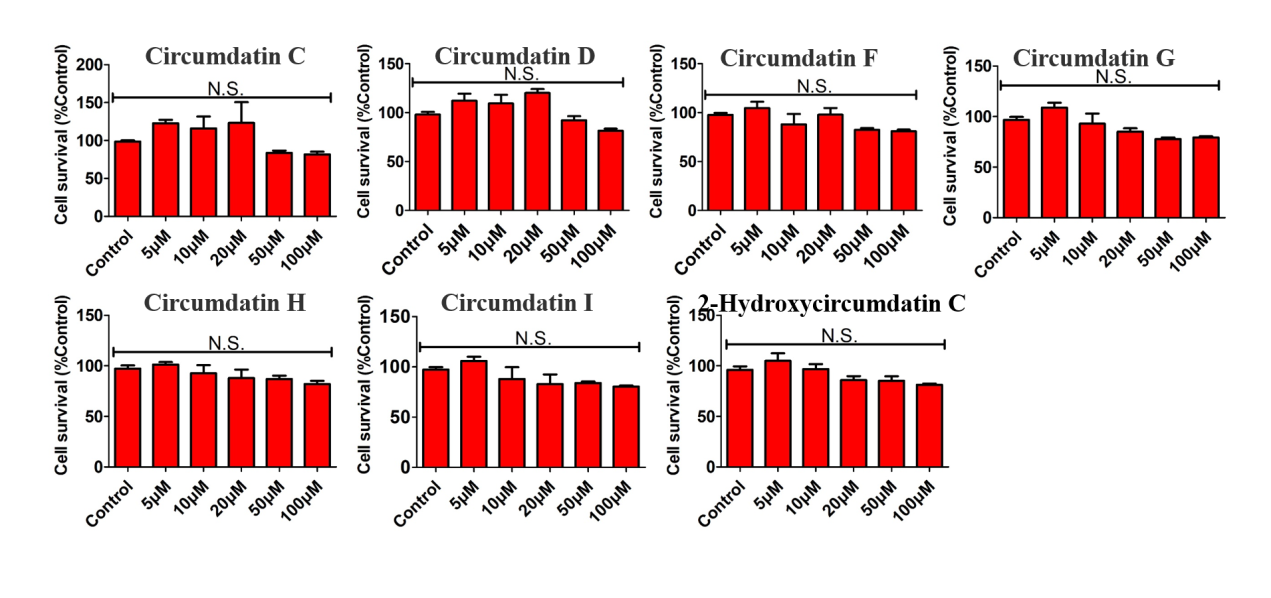
Figure S16** Effects of circumdatins on cell survivability in BV-2 cells.

Cellular survivability was assessed using the MTT assay. BV-2 cells (5 × 10^3^ cell/well) were treated with the suggested concentrations of circumdatins for 48 hours. The results shown are mean ± SD and represent three independent tests. N.S. means no significant differences from the control cells.

**
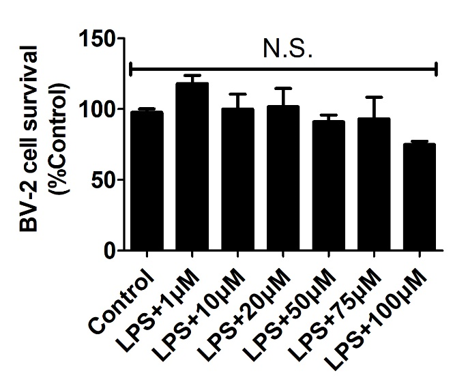
**

**Figure S17** Effects of circumdatins D on cell survivability in BV-2 cells.

BV-2 cells (5 × 10^3^ cell/well) were stimulated with 1μg/mL LPS with or without circumdatin D (1-100μM) for 48 h by MTT assay(a，b) . The results shown are mean ± SD and represent three independent tests. N.S means no significant differences from the control cells.

**
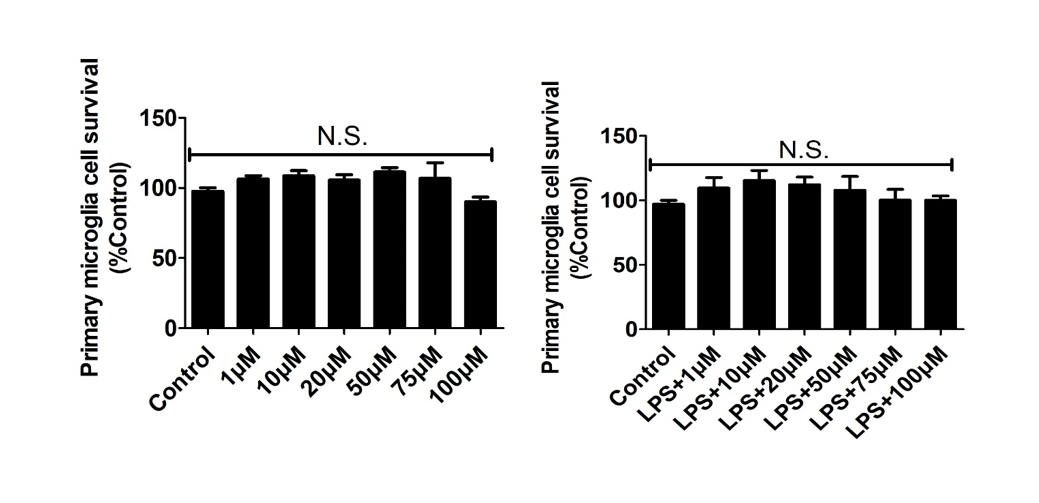
**

**Figure S18** Effects of circumdatins D on cell survivability in primary microglia cells. Primary microglia cell (1 × 10^4^ cell/well) was stimulated with or without 1μg/mL LPS in the presence or absence of circumdatin D (1-100μM) for 48 h by MTT assay(a，b) . The results shown are mean ± SD and represent three independent tests. N.S. means no significant differences from the control cells.

**
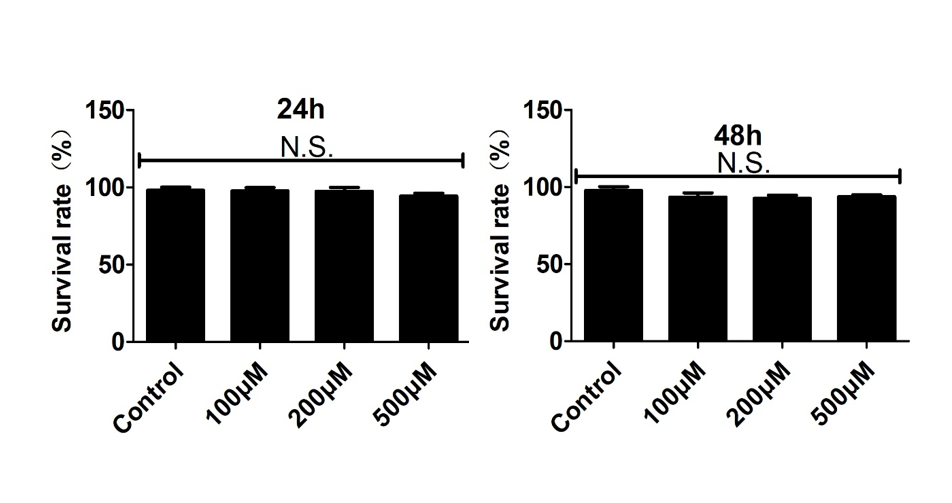
Figure S19** The toxicity of circumdatin D on CL4176 nematodes in 24h and 48h.

*C. elegans* strains CL4176 was exposed to varying concentrations of circumdatin D (50μM、100μM、200μM) for indicated time. The worms were considered as death if there was no response when gently touched with a platinum wire. Data were from three independent assays with 60 worms each and presented in mean ± SD. N.S. means no significant differences from the vehicle-treated group.

**
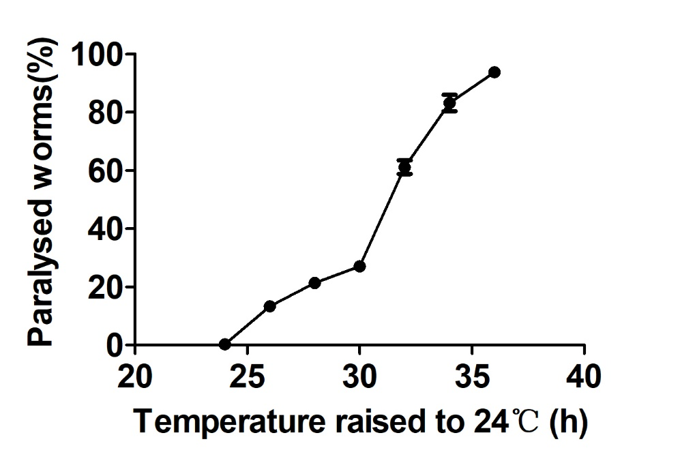
**

**Figure S20** Optimization of experimental conditions in paralysis assay.

CL4176 worms were egg-synchronized onto fresh NGM plates containing OP50 bacteria at 16℃. The temperature was then raised to 25 °C, and the number of paralyzed worms was recorded at 24, 26, 28, 30, 32, 36h under the microscope, respectively. The worms were considered as paralyzed if there was no response or they only moved their head when gently touched with a platinum wire. Data were from three independent assays with 90 worms each and presented in mean ± SD. Error bar represents SD.

**References**

Cui, C., Li, X., Li, C., Sun, H., Gao, S., Wang, B. (2009) Benzodiazepine alkaloids from marine-derived endophytic fungus Aspergillus ochraceus. Helv. Chim. Acta. 92: 1366-1370.

Dai, J., Carte, B.K., Sidebottom, P.J., Yew, A.L.S., Ng, S.B., Huang, Y., Butler, M.S. (2001) Circumdatin G, a new alkaloid from the fungus Aspergillus ochraceus. J. Nat. Prod. 64: 125-126.

López-Gresa, M.P., González, M.C., Primo, J., Moya, P., Romero, V., Estornell, E. (2005) Circumdatin H, a new inhibitor of mitochondrial NADH oxidase, from Aspergillus ochraceus. J. Antibiot. 58(6): 416-419.

Rahbæk, L., Breinholt, J. (1999) Circumdatins D, E, and F: further fungal benzodiazepine analogues from Aspergillus ochraceus. J. Nat. Prod. 62: 904-905.

Zhang, D., Yang, X., Kang, J.S., Choi, H.D., Son, B.W. (2008) Circumdatin I, a new ultraviolet-A protecting benzodiazepine alkaloid from a marine isolate of the fungus Exophiala. J. Antibiot. 61(1): 40-42.

Ookura, R., Kito, K., Ooi, T., Namikoshi, M., Kusumi, T. (2008) Structure revision of circumdatins A and B, benzodiazepine alkaloids produced by marine fungus Aspergillus ostianus, by X-ray crystallography. J. Org. Chem. 73: 4245-4247.

Rahbæk, L., Breinholt, J., Frisvad, J.C., Christophersen, C. (1999) Circumdatin A, B, and C: three new benzodiazepine alkaloids isolated from a culture of the fungus Aspergillus ochraceus. J. Org. Chem.64: 1689-1692.
